# Microwave Ultrafast Heating on Iron‐based Nanogap Catalysts for CO_2_ Reduction Coupling with Coke Removal

**DOI:** 10.1002/advs.202518411

**Published:** 2025-12-25

**Authors:** Xi Shen, Zhenyu Zhao, Jinsong Zhang, Hong Li, Xin Gao

**Affiliations:** ^1^ School of Chemical Engineering and Technology National Engineering Research Center of Distillation Technology Collaborative Innovation Center of Chemical Science and Engineering (Tianjin) Tianjin University Tianjin 300350 China; ^2^ Institute of Structured and Architected Materials Liaoning Academy of Materials Shenyang 110167 China; ^3^ Haihe Laboratory of Sustainable Chemical Transformations Tianjin 300192 China

**Keywords:** CO_2_ reduction, coke removal, microwave, net‐zero carbon factory, plastic pyrolysis

## Abstract

The persistent challenge of catalytic coke deposition is compounded by conventional regeneration methods like air combustion that are both energy‐intensive and emit additional CO_2_, contradicting global carbon neutrality objectives. Although using CO_2_ to convert coke into CO offers a route to simultaneous catalyst regeneration and CO_2_ reduction, its application is constrained by the weak oxidizing capacity of CO_2_, especially toward highly graphitized cokes. Herein, we develop a microwave‐driven technique that enables efficient coke conversion by anchoring iron nanoparticles onto the coke surfaces within seconds. These nanoparticles can not only catalyze the Boudouard reaction but also construct abundant nanogaps that locally enhance electromagnetic fields, resulting in extreme heating to 1990.4 K while maintaining reactor wall temperatures below 800 K. This approach eliminates the need for extremely thermal‐resistant reactor material and fully reactivates iron‐based catalysts for cyclic plastic pyrolysis. Moreover, the residual coke after treatment evolves into low‐defect CNTs (I_D_ /I_G_ = 0.192) outperforming commercial counterparts. Techno‐economic and carbon footprint analyses confirm the feasibility of a net‐zero emission plant, in which captured CO_2_ from utility systems is recycled as an oxidant for coke removal, offering a sustainable pathway for coke management and carbon utilization in chemical industries.

## Graphical abstract

This study employs a nanogap‐induced microwave discharge strategy to pretreat coke deposits on iron‐based catalysts with engineered nanogaps, causing ultrarapid heating to 1990 K within seconds, selectively eliminating >70% surface carbon deposits and converting waste plastics and greenhouse gases into syngas. The integration of ultrafast pyrolysis, catalyst recycling, and carbon management advances a microwave‐driven net‐zero economy.

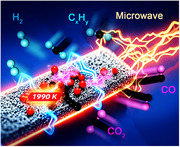

## 1 Introduction

Metallic‐based catalysts are widely applied in high‐temperature reactions [1], such as waste biomass/plastic pyrolysis [2], methane dry reforming [3], alkane dehydrogenation [4], Fischer‐Tropsch synthesis [5], etc. However, catalyst reactivation always occurs mainly because carbon deposits accumulated on catalyst particles prevent the reactants from reaching the active sites, representing a pervasive industrial challenge [6]. The deactivated catalysts must be regenerated by air combustion (C + O_2_ → CO_2_) or steam gasification [7] to eliminate carbon deposits, which not only requires additional energy consumption but also emits extra CO_2_. The increasing demand for carbon neutrality calls for a novel regeneration strategy to directionally transform coke to resources [8]. The employment of CO_2_ as the oxidative for high temperature reactions (e.g., propane dehydrogenation [9], methane reforming [10]) not only reduces the deactivation rate of catalysts, but also achieves CO_2_ emission reduction and resource recovery. In the above findings, CO_2_ is only utilized as a soft oxidant for suppressing the formation of coke, rather than acts as the reactant to directly transform carbons to carbon oxide via the Boudouard reaction (C + CO_2_ ⇌ 2CO), mainly because the inherent kinetic limitations of CO_2_ activation demand temperatures above 1200 K for graphitized coke (e.g, carbon nanotubes from plastic pyrolysis), giving rise to an intractable dilemma between temperature rise demand and reactor materials durability.

Microwave technology has emerged as a process intensification alternative to resolve the fundamental thermal paradox between elevated operating temperatures and reactor material tolerance [11], because strong absorbing substrates can be heated to extremely high temperatures with dramatic heating rate (above 2000 K/min [12]) under microwave irradiation, which can overcome the heating limitations in conventional heating methods, rendering microwave‐assisted process reduced time and energy consumption [13, 14, 15, 16, 17]. Besides, the selective heating characteristic of microwave irradiation enables the direct energy delivery to target substances, resulting in the formation of localized superheating at microscopic scales [18]. Our previous study has quantitatively mapped this “hot spots” phenomenon using in situ fluorescence thermometry [19, 20, 21], revealing that the thermal gradient at the overheating domains is not only determined by the electromagnetic field intensity, but also dependent on microwave absorbing capacity (i.e., dielectric loss). Coincidentally, graphitic coke intrinsically functions as an efficient microwave susceptor [22, 23, 24], because under a rapidly alternating electromagnetic field, the collision between periodically moved electrons and carbon atoms in the polarized region leads to Joule current and therefore ultrafast temperature rise [25]. This unique nonequilibrium energy conversion route happens in the nanosized space of carbon materials, while the external environment can still be maintained at mild temperatures [26]. Therefore, the above inherent synergy endows microwave technology with a natural advantage in the field of carbon deposition treatment.

To achieve extremely high temperatures to reach Boudouard reaction thresholds and minimize thermal dissipation to reactor walls, it is still necessary to pretreat carbon deposits to improve their microwave absorbing efficiency, owing to the microwave‐reflective characteristic of graphitic coke with high electrical conductivity [27]. Currently, the approaches to preparing strong microwave‐absorbing carbon materials mainly include synergy of multiple microwave adsorption mechanisms and rational construction of microstructures [28]. The former indicates the combination of dielectric loss resulting from electrical conductivity and polarization with other possible loss mechanisms (e.g., magnetic loss) by synthesizing metal‐carbon heterostructures to achieve excellent impedance matching [29]. The latter refers to the fabrication of nano‐voids or gaps that enable the multiple reflection and scattering paths of electromagnetic waves inside the material [30]. In principle, the formation of nanogaps between conductive surfaces can strengthen localized electromagnetic fields, thereby enabling rapid temperature rise under microwave irradiation [31, 32]. For example, the gaps between crosslinked conductive carbon fibers can trigger a rapid temperature rise with a ramp rate exceeding 300°C/s under microwave irradiation [33], facilitating the preparation of CuO/carbon fiber composites for electrocatalytic applications. Similarly, by engineering microstructural defects within a carbon matrix at the nanoscale, the graphene nanosheets exhibited significantly higher local heating rates, forming an electrocatalyst for CO_2_ reduction [34].

**FIGURE 1:**
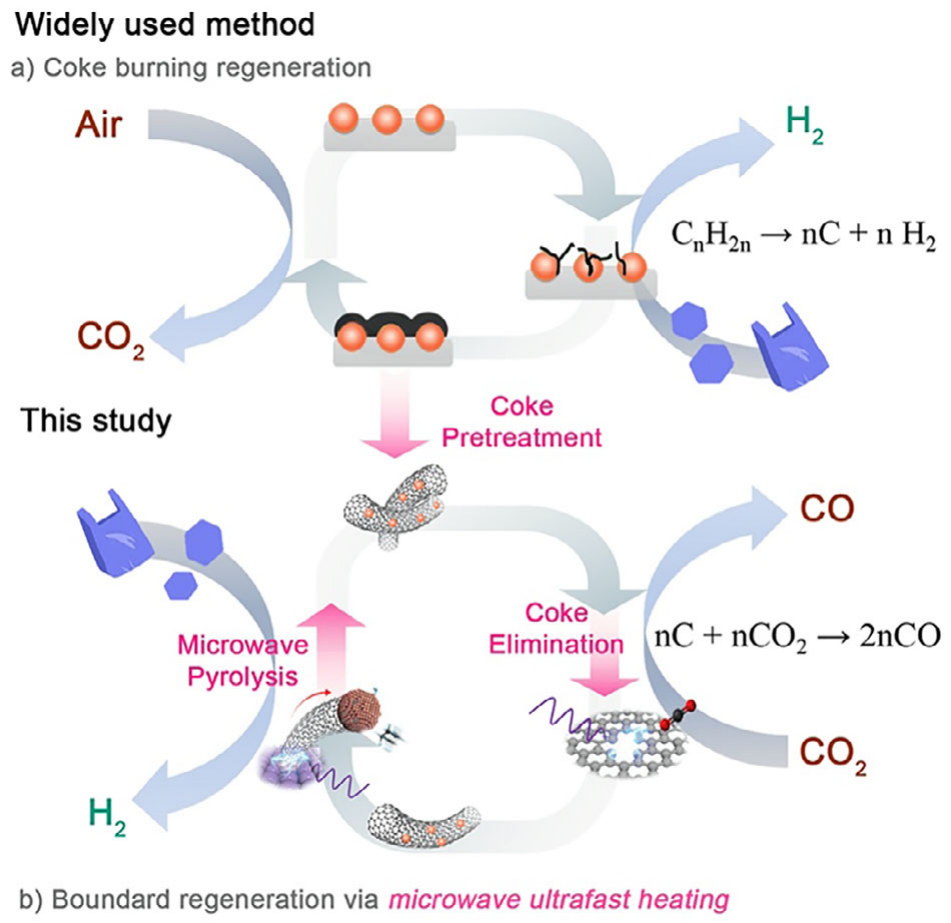
Graphical illustration of the role of microwave‐assisted technologies used in this study, including (1) coke pretreatment to deposit finely dispersed iron nanoparticles onto the coke surface, (2) coke removal through initiating the Boudouard reaction via instantaneous superheating, and (3) rapid pyrolysis of plastic wastes to bypass undesirable low‐temperature reaction pathways.

Based on the application scenarios and process requirements for coke deposition treatment of iron‐based catalysts, the present study uses highly graphitized coke generated from iron‐catalyzed plastic pyrolysis (a representative that is rather difficult to remove) as the coke model system, on which iron nanoparticles with high number density are deposited within seconds through a microwave‐induced decomposition route, as shown in Figure 1. These nanoparticles can not only catalyze the Boudouard reaction, but also result in an instantaneous rise of localized temperature to thousands of degrees, enabling efficient coke elimination and CO_2_ reduction. Based on this novel coke elimination approach, we propose a concept of a net‐zero carbon factory by integrating plastic pyrolysis with CO_2_ induced coke removal. Within the plant, CO_2_ emissions from utility steam generation and power production during chemical manufacturing are captured and reused as feedstock for microwave‐induced catalyst regeneration. The resulting carbon monoxide and high‐value carbon materials after purification are used as chemical materials, while the unreacted CO_2_ is recovered for subsequent usage in catalyst regeneration, thereby achieving net‐zero carbon emissions for the entire facility.

## 2 Results and Discussion

### 2.1 Coke Pretreatment for Extreme Microwave Heating

**FIGURE 2:**
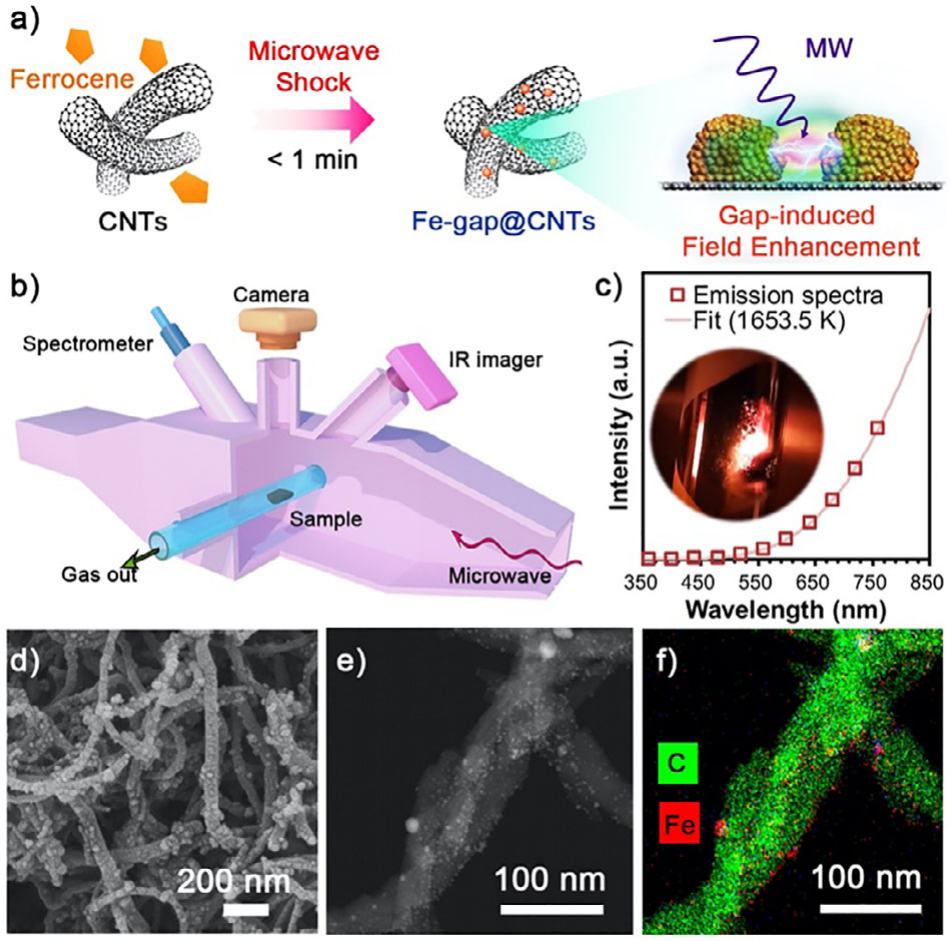
Coke pretreatment via a microwave‐induced ferrocene decomposition to anchor iron particles on its surface. (a) Schematic diagram of the iron anchoring process and the principle of nanogap‐induced rapid heating. (b) Schematic diagram of the microwave cavity utilized for the microwave‐induced arc discharge process. (c) Emission spectrum and fitted sample temperature during Fe anchoring. (d) SEM image, (e) STEM image, and (f) Elemental distribution of coke samples after iron anchoring.

Here, the model carbon deposits used for coke elimination experiments were carbon nanotubes (CNTs), because this model system represented one of the most graphitized and chemically stable forms of coke carbon commonly encountered in catalytic processes, as the formation of CNTs on catalyst surfaces was often a critical deactivation pathway via encapsulating and blocking the active sites [35]. Here, we employed a “worst‐case scenario” strategy by demonstrating efficacy against the most challenging coke, which was obtained through microwave pyrolysis of polypropylene (PP) catalyzed by FeAlOx that as reported in our previous study [36]. The detailed experimental procedures of microwave‐assisted pyrolysis are described in Section S1 (Figures S1–S3). The morphology of solid residues is characterized via SEM and TEM analysis (Figure S4), demonstrating that the iron‐based catalyst is covered by the deposited carbon that exists in the form of CNTs with an average diameter of 29 nm. The obtained coke substrate is pretreated by a microwave‐induced arc discharge technique [37] to induce rapid high‐temperature decomposition of metal organic precursors, introducing nanoparticles anchored on the surface of CNTs, as illustrated in Figure 2a. The substrate was blended with ferrocene, and the mixture was loaded into the microwave tubular reactor as shown in Figure 2b (Details of experimental procedures are demonstrated in the section S1.3. The microwave energy absorbed by iron‐based catalyst particles induces micro‐discharge between iron nanoparticles due to the field‐strengthening effect, which is continuously recorded in real time using an HD camera through a viewport at the top of the cavity. Within seconds of microwave initiation, intense scintillating discharge phenomena occurred inside the reaction tube, and the sample emitted a dazzling yellow light (Video S1). The latter phenomenon can be ascribed to the blackbody radiation of high‐temperature samples. Considering that the intensity of spectral radiation is only determined by the sample's temperature (Planck's law of blackbody radiation), we here calculate the temperature of the microwave‐irradiated sample by fitting the emission spectra obtained from the spectrometer installed on the cavity's viewing window, where the detailed calculation method is described in Section S2.1. The fitted results are confirmed by a colorimetric thermometry approach developed in this study (Figure S5), indicating the instantaneous sample heating to 1653.5 K within tens of seconds (Figure 2c).

Consistent with our previous reports [38], the rapid local heating in a short period of time causes ferrocene to decompose rapidly, avoiding its massive volatilization and enabling the produced iron nanoparticles to anchor on the surface of carbon deposits, which is evidenced by the increase of iron content from 5.0 to 10.6 wt.% in the coke substrates (ICP‐MS measurement in Figure S6). Compared to the original stacked carbon nanotubes (SEM images in Figure S4), the CNTs substrate pretreated by microwave‐induced ferrocene decomposition is decorated with numerous fine nanoparticles, as demonstrated in the SEM images (Figure 2d). These nanoparticles primarily exist in the form of metallic iron, according to the XRD analysis (Figure S7). Besides, XRD patterns of solid residues obtained by various cycles of PP pyrolysis exhibit a significant increase in the intensity ratio of the peak at 2θ = 26° to that at 2θ = 45° after five cycles of PP pyrolysis, confirming the formation of carbon deposits due to CNTs accumulation that dilutes the iron concentration in solid residues. STEM images and elemental mapping (Figure 2e,f) show that the sizes of most iron nanoparticles in the range 1.5–3 nm, whose size distribution is demonstrated in Figure S8 in the Supporting Information. These tiny nanoparticles are arranged in a matrix, with a number density of more than 300/(100 nm)^2^ (Figure S9).

### 2.2 Coke Elimination at Localized Ultrahigh Temperatures

**FIGURE 3:**
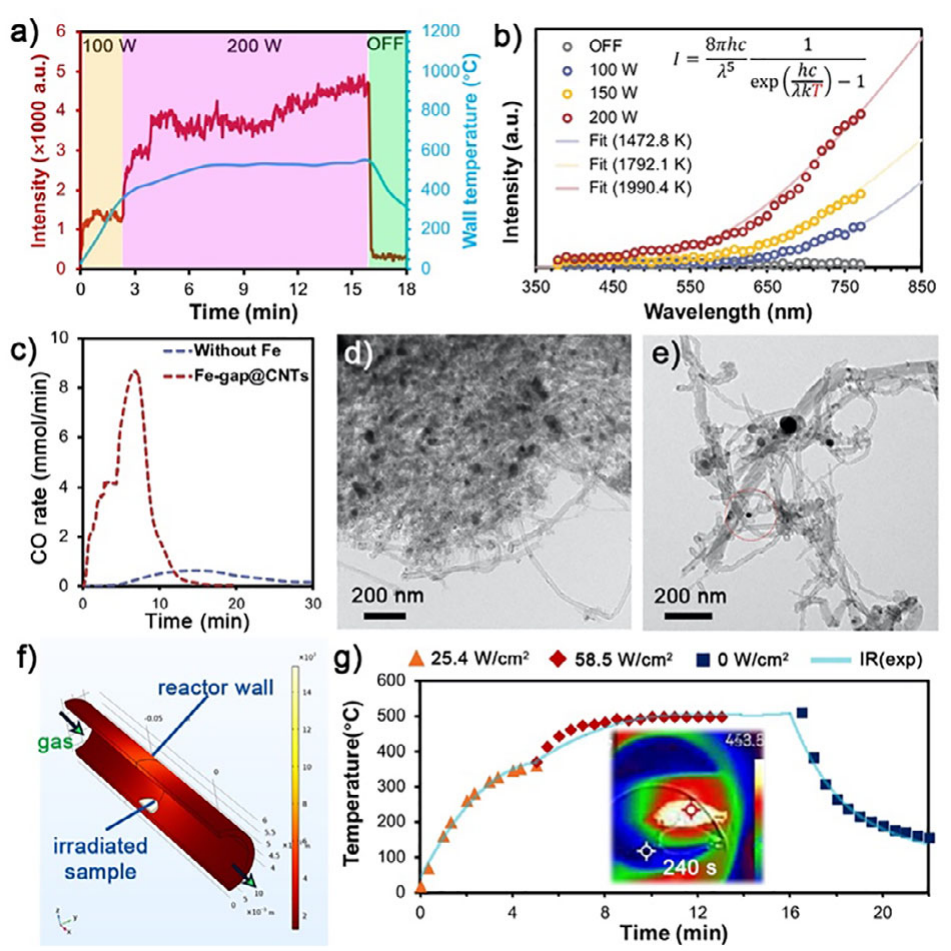
Coke removal at CO_2_ atmosphere under microwave‐induced ultrahigh temperatures. (a) Time‐varying intensity of luminescence (830 nm) emitted from the sample and the temperature of the tube reactor wall. (b) Estimation of the sample temperature under microwave irradiation based on Planck's black‐body theory. (c) Generation rate of CO during microwave irradiated coke removal in the presence/absence of iron nanoparticles. (d) TEM imaging of solid residues after microwave‐assisted pyrolysis of PP catalyzed by iron‐based catalysts. (e) TEM imaging of solid residues after microwave‐induced coke elimination. (f) Numerical simulation of thermal radiation emitted from the sample at elevated temperatures. (g) Simulated results and IR measurements of reactor wall temperature heated by the sample's thermal radiation.

After depositing iron nanoparticles on the CNTs surfaces, we turned off the microwave generator to allow the sample to cool down. Subsequently, CO_2_ was introduced into the microwave tubular with a volumetric flow rate of 200 mL/min to establish the oxidation reaction atmosphere. The exhaust gas from the reactor was directed to a gas analyzer to enable real‐time monitoring of the reaction composition. Upon activating the microwave generator at 100 W, intense red luminescence emitted from the sample was detected by the camera (Figure S10) and the photoluminescence spectrometer within seconds (Figure 3a). Spectral fitting of the luminescence showed that the sample temperature reached 1472.8 K (Figure 3b). When the microwave power was increased to 200 W, the luminescence area of the sample expanded, with the luminescence color shifting from dark red to orange red (Figure S10). This phenomenon should be ascribed to the blueshift of blackbody radiation as a result of temperature increase, where the sample temperature was determined to be 1990.4 K according to the spectral fitting. The progress of coke elimination can be monitored by the CO generation detected in the exhaust gas (Figure 3c), demonstrating that enlarging the microwave power at the third min accelerates the Boudouard rate and therefore increases the instantaneous CO concentration, which begins to decline after reaching its peak at the 8th min. Finally, the reaction ended within 15 min, leading to the decrease of sample mass by 62 wt.% (Figure S11). This finding indicates that above 73% of deposited carbon is removed, considering that the weight of iron‐containing particles accounts for 35.7 wt.% of the total mass of the solid residues. In contrast, the coke substrate in the absence of iron nanoparticles under the identical microwave radiation conditions demonstrated slower heating to the target temperature and with lower CO production compared to Fe‐gap@CNT, where only 15% of deposited carbon is eliminated. Compared to pre‐reaction coke samples showing dense encapsulation of iron nanoparticles with CNTs (TEM image in Figure 3d), the solid residues after microwave‐induced carbon elimination in Figure 3e demonstrate substantial degradation of the CNTs, exposing the catalysts for potential reuse in plastic pyrolysis.

Notably, despite the sample reaching 1990.4 K (Figure 3b) in the process of microwave‐induced coke elimination, the reactor wall only remained below 800 K, where the temperature of the tubular reactor wall is monitored by an IR camera (Hikvision, HM‐TD2037T‐4/X) as shown in Figures S12 and S13. This temperature difference is mainly attributed to the microwave‐transparent property of the quartz material, preventing the reactor wall to be heated by microwave. Its temperature rise originates primarily from thermal radiation emitted by the high‐temperature sample. To quantitatively validate this selective heating characteristic of microwave, a numerical simulation model is conducted by using COMSOL Multiphysics, as shown in Figure 3f. The details of boundary conditions and key parameters are described in Section S4 of simulation, including Figures S14–S17 and Tables S1 and S2). When a 10‐mm diameter circular sample was heated to 1472.8 K, it radiated heat to the environment at an intensity of 25.4 W/cm^2^, causing the quartz wall at 10 mm distance to heat at a ramp rate of 150 K/min and reach thermal equilibrium at 633 K in approximately 4 min under forced cooling by flowing air outside the reactor, as shown in Figure 3g. Upon increasing the sample temperature to 1990.4 K, the radiative power rose to 58.5 W/cm^2^, elevating the equilibrium temperature of the reactor wall to 778 K. This simulation result showed excellent agreement with the infrared thermography observations, quantitatively confirming the localized superheating effect during microwave‐induced coke elimination. This effect allows the Boudouard reaction to be operated under relatively mild conditions, avoiding the requirement of extreme equipment specifications.

### 2.3 Dual Functionality of Anchored Iron Nanoparticles

**FIGURE 4:**
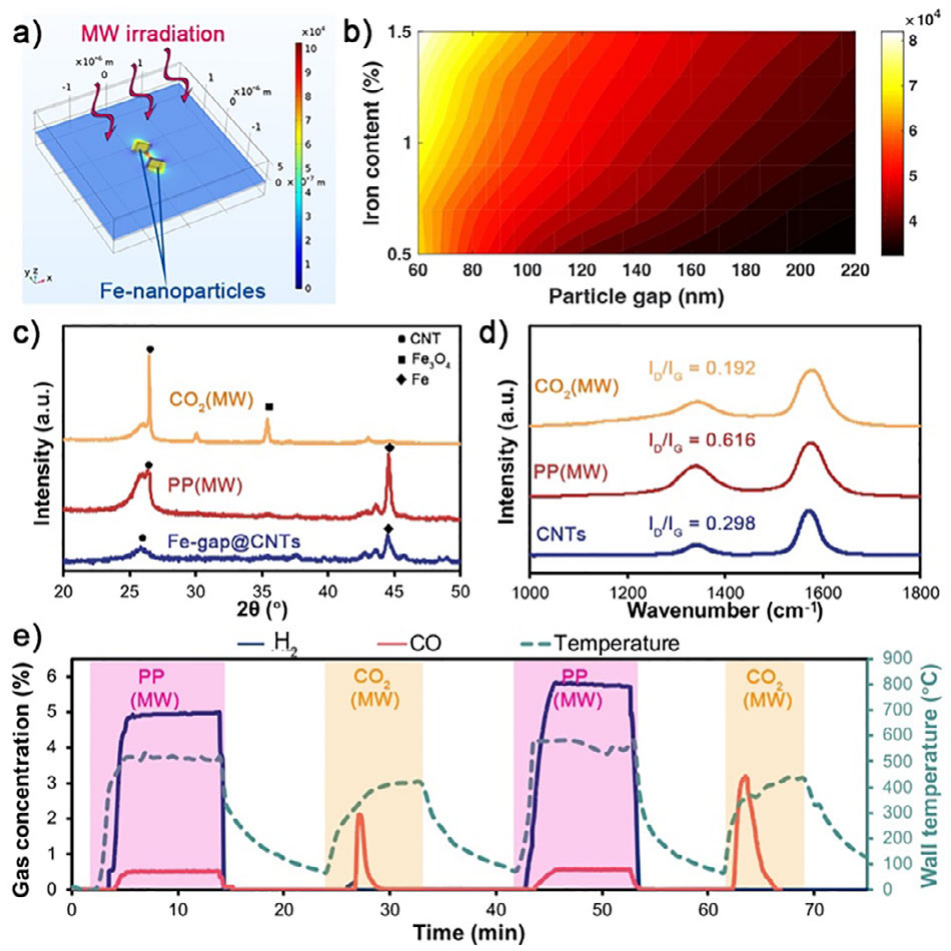
Dual functionality of anchored iron nanoparticles. (a) Numerical simulation of electrical field enhancement induced by nanogaps. (b) Influence of gap distance and iron content on electrical field enhancement. (c) XRD patterns and (d) Raman spectra of solid residue after one‐cycle PP pyrolysis and CO_2_ coke elimination. (e) Temporal evolution of gas concentration and tube wall temperature during the pyrolysis‐coke elimination shift catalyzed by Fe‐gap@CNT.

Understanding the mechanism of ultrafast heating is critical for further designing microwave responsive catalysts towards the Boudouard reactions with improved energy efficiency. Therefore, we employed a COMSOL Multiphysics numerical model at the microscale to simulate the electromagnetic field concentration effect, as illustrated in Figure 4a. The model incorporated a conductive 2D plane representing the highly graphitized coke, with rounded‐square particles on the coke surface mimicking iron nanoparticles. Electromagnetic waves are introduced via a wave optics port to analyze the field distribution, where the detailed calculation conditions are described in Section S5, including Figures S19–S21 and Tables S3 and S4. Simulation results revealed that the electromagnetic field intensity at the particle gaps was significantly higher than the average level. Moreover, parametric sweep analysis (Figure 4b), varying particle quantity and spacing showed that a 1.5‐fold increase in the content of conductive particles raised the maximal electromagnetic field intensity by 10.6%, while this value reached 26.7% when the particle content was increased to 2.25 times relative to the initial value. For the same content of conductive particles, reducing particle size can lead to an increased number density of iron particles and narrower interparticle spacing. According to the simulation results, the decrease of particle spacing by to 27% of its original value can nearly double the electromagnetic field intensity at the gaps. This field enhancement effect is also confirmed in the preparation of coke substrates during microwave‐assisted pyrolysis. After being treated with 2300 K thermal shock on carbon paper, Fe_3_O_4_ nanocrystals seep out on the surface of amorphous FeAlOx, as evidenced by SEM images in Figure S22 and XRD patterns in Figure S23, resulting in a dramatic increase in its microwave absorbing capability with a heating rate of 368 K/min under 200 W microwave irradiation (Figure S24). The ultrafast heating avoids prolonged residence time in the low‐temperature regime, decreasing the generation of wax compared to conventional Fe‐based catalysts (Figure S25). Nevertheless, the increase in pyrolysis cycle number reduces the iron content (Schematic illustration in Figure S26 and ICP‐MS results in Figure S6) and enlarges the spacing between iron particles due to the growth of CNTs (SEM scanning in Figure S27). The reduction of iron particles’ number density significantly decreases the microwave heating rates and even causes the failure of microwave induced ultrafast heating (Figure S28), which agrees with the simulation discussion in Figure 4b.

In microwave‐induced coke elimination within a CO_2_ atmosphere, the iron particles anchored on CNTs can not only trigger ultrafast overheating, but also serve as catalysts for the reaction between CNTs and CO_2_. The surface of highly graphitized carbon lacks adsorption sites for CO_2_, hindering the proceeding of the Boudouard reaction. During the process of coke elimination, the numerous iron nanoparticles (Fe^0^) deposited on CNTs were oxidized to Fe_3_O_4_ (XRD results in Figure 4c) in a CO_2_ atmosphere. The resulting Fe_3_O species are highly active for gasifying surrounding graphitic coke to CO, establishing a dynamic Fe^0^ ↔ Fe_3_O_4_ redox cycle. This cycle boosted the coke conversion efficiency by more than 4‐fold compared to the case in the absence of anchored Fe nanoparticles (Figure S11). Notably, after coke elimination reactions, the characteristic peak at 2θ = 26° (MWCNT) became much sharper, which can be attributed to the CO_2_’s preference for reacting with carbon at defect sites, leaving only low‐defect CNTs unreacted after coke elimination reactions. The formation of a highly graphitized structure with long‐range order can be confirmed by the Raman analysis (as exhibited in Figure 4d). The defect degree (I_D_ / I_G_ ratio) of plastic pyrolysis‐derived coke decreased from 0.616 to 0.192 in the remaining sample after CO_2_ treatment, which is even lower than commercial CNTs. XPS further demonstrates a dominant sp^2^‐hybridized carbon signature in the C 1s spectrum, with minimal contributions from sp^3^ carbon or oxygen‐containing groups (Figure S29), revealing the chemical purity and structural integrity of the obtained material. The low defect characteristic was also validated by complementary temperature‐programmed oxidation (TPO) profiles, as shown in Figure S30. The CO and CO_2_ evolution peaks, as well as the O_2_ consumption profile, shift toward higher temperatures compared to pristine Fe‐CNTs. The low‐defect structure endowed CNTs with higher electrical conductivity (44.114 S/cm) under an applied pressure of 30 MPa (Figure S31), even much larger than commercial products (36.406 S/cm). Therefore, raw materials with superior electrical properties can be provided for the field of electronics and energy storage [39, 40] by conducting microwave‐initiated carbon treatment.

In a practical system for continuous plastic‐to‐hydrogen conversion, the catalyst should be periodically regenerated to maintain its catalytic activity by removing accumulated coke. The intrinsic catalytic activity and microwave susceptor property endow Fe‐gap@CNTs with repeated applicability in microwave‐assisted pyrolysis of waste plastics, facilitating the establishment of a closed‐loop conversion from greenhouse gas and waste PP plastic to syngas, designated as CLC‐CO_2_&PP, as demonstrated in Figure S32. The iron nanoparticles act as localized microwave susceptors, generating extreme heating that drives both the Boudouard reaction and plastic dehydrogenation, thereby continuously producing hydrogen and CO‐rich gas. Over multiple pyrolysis‐coke removal cycles, CNTs with low defects are likely to accumulate, as they are resistant to gasification by CO_2_. Then these residual carbon species not only serve as conductivity supports but also can be reactivated into dual‐function catalysts via ferrocene decomposition pretreatment, making them suitable for reuse in subsequent CLC‐CO2&PP. To confirm the feasibility of the CLC‐CO_2_&PP route, we reused Fe‐gap@CNT as a catalyst for the pyrolysis of waste PP under conditions consistent with Section S1.3. Under microwave irradiation, significant luminescence was also observed (Video S2), converting PP into hydrogen (Figure 4e) and carbon nanotubes (evidenced by XRD patterns in Figure 4d and SEM images in Figure S33) within minutes. Simultaneously, since metallic iron (Fe^0^) nanoparticles were oxidized to Fe_3_O_4_ during the CO_2_‐based coke elimination process (Figure 4d), minor CO byproducts were generated during PP pyrolysis, as exhibited in Figure 4e. Similar to the coke removal process, although the sample temperature reached 1472 K (the fitted results of emitted luminescence are exhibited in Figure S34), the reactor wall temperature remained below 653 K (IR measurement results are exhibited in Figure S35). Moreover, due to extremely rapid heating achieved by nanogap‐induced local intensification of electromagnetic field, no oil or wax products were observed during microwave pyrolysis catalyzed by Fe‐gap@CNTs (Figure S36), eliminating the complex separation processes for purifying hydrocarbons that are always generated in previously reported pyrolysis processes [36, 43, 44]. After plastic pyrolysis, switching the atmosphere to CO_2_ removed newly deposited coke on the catalyst surface by converting it into CO. The CLC‐CO_2_&PP system maintained excellent stability across multiple switching cycles (Figure 4e). To validate the applicability of this strategy, real industrial carbon waste was employed to repeat the microwave heating process, exhibiting that microwave‐induced ultrahigh temperatures enable rapid conversion of such carbon wastes (less stable than CNTs) in a CO_2_ atmosphere within minutes (Figure S37).

### 2.4 Economic and Carbon Emission Analysis

**FIGURE 5:**
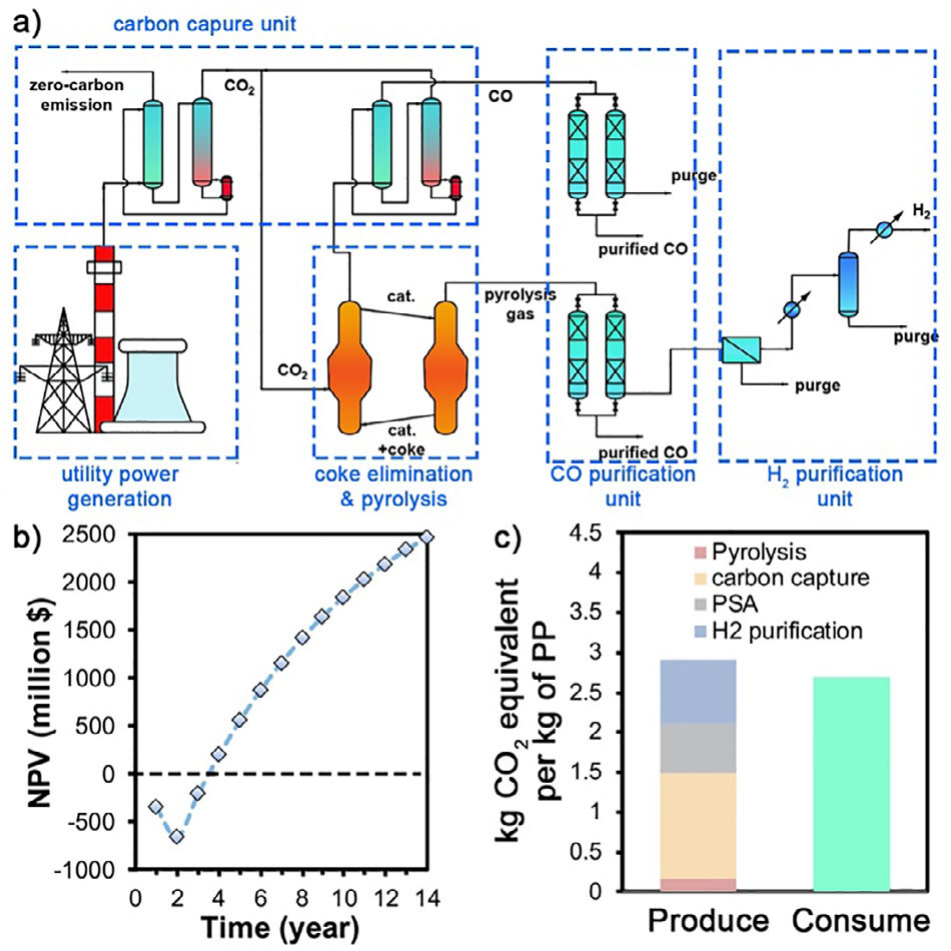
Economic and CO_2_ emission analysis of coke elimination integrated with microwave‐assisted waste PP pyrolysis. (a) Schematic diagram of the unit operations in the zero‐carbon factory. (b) The static payback period of this technology model (100 kton/a PP). The construction period was set to 2 years. (c) The greenhouse gas emissions and consumption from treating 1 kg of PP wastes.

The CO_2_ reduction method for coke treatment was integrated into the microwave pyrolysis of PP for hydrogen production, refining the zero‐carbon plant concept illustrated in Figure 5a. The plant comprises three interconnected sections, involving a coke elimination unit, a carbon capture unit, and purification units for CO and H_2_. Emissions of CO_2_ generated during fuel combustion for electricity and steam requirements are managed with the carbon capture system, where the captured CO_2_ is recycled as a reactant for the coke elimination unit, after which the unreacted CO_2_ in the tail gas is reintroduced to the carbon capture unit for collection and reuse. Treated gas from the coke elimination unit is directed to a pressure swing adsorption (PSA) system to capture the generated CO and remove the impurities. The purified CO serves as a feedstock for syngas production, enabling the synthesis of basic chemical products. Additionally, a secondary PSA system recovers CO from hydrogen‐rich gases generated during plastic pyrolysis. The treated gas is directed to a hydrogen purification section, where a membrane separation coupled with cryogenic distillation is employed to maximize hydrogen yield, achieving higher recovery rates compared to PSA despite higher energy consumption [45]. Low‐defect CNTs remaining after CO_2_‐mediated coke elimination are partially sold as high‐value products, while the remainder is supplemented with iron via the ferrocene decomposition method and reintroduced to the cycle of plastic pyrolysis‐CO_2_ reduction. Detailed economic assessments of the process are provided in Section S7, including Tables S5–S10. For a 100 000 tons/y facility of PP plastic pyrolysis and CO_2_ reduction, initial investments primarily target microwave equipment and reactor construction, while operational costs are dominated by energy consumption associated with product separation (Table S8). Energy use for carbon capture, CO purification, and H_2_ purification accounts for 80.9% of the total process consumption, with cryogenic distillation refrigeration alone contributing 74% of this expenditure. Despite these energy demands, the market value of CO and CNTs products (Table S7) ensures that the process to achieve a positive net present value (NPV) by the fourth year of operation, as illustrated in Figure 5b. Carbon capture in this study employs mature monoethanolamide (MEA) absorption technology, requiring approximately 4 GJ of steam per ton of CO_2_ captured for regeneration of the absorbent. The production of steam leads to annual CO_2_ emissions of at least 132 000 tons. Additional CO_2_ emissions from power generation for CO and H_2_ purification reach 62 500 and 79 200 tons, respectively. Overall, processing 1 kg of waste PP generates 2.75 kg of CO_2_ emissions, of which 2.69 kg CO_2_ can be converted to CO through the microwave induced coke elimination process, indicating an essential balance of CO_2_ emissions, as exhibited in Figure 5c. These findings validate the feasibility of constructing a zero‐carbon plant using the proposed microwave‐induced coke removal method. Future implementation of process intensification technologies in gas separation and purification sections, particularly in hydrogen purification, can further reduce energy consumption and lower the plant's overall carbon emissions, potentially enabling negative‐carbon factory operations.

## 3 Conclusion

This work introduces a transformative microwave‐driven strategy for sustainable carbon management by converting detrimental coke deposits into functional electromagnetic nanoreactors via in situ deposition of high‐density iron nanoparticles. The interparticle gaps leads to a 5–7‐fold enhancement of local electromagnetic fields, enabling ultrafast heating at rates up to 11,057 K/min and to reach 1990.4 K under a low microwave power of 200 W. Crucially, this localized superheating is confined to the coke region, maintaining the bulk reactor temperature below 800 K, thereby bypassing the material limitations of conventional high‐temperature reactors and minimizing energy waste associated with global heating. Apart from the capability of inducing ultrafast overheating, these iron nanoparticles catalytically promote the Boudouard reaction, enhancing the CO_2_ reduction kinetics by 12.3‐fold compared to unmodified coke. This reaction selectively removes disordered carbon, resulting in high‐product CNTs with an I_D_ /I_G_ ratio of 0.192, outperforming commercial CNTs that are suitable for energy and conductive applications. Techno‐economic analysis and carbon footprint analyses validate the scalability of this strategy for a net‐zero emission plant. Integrated with plastic pyrolysis, the process converts 2.69 kg CO_2_ per kg of waste polypropylene into high‐purity CO. Despite 80.9% of operating costs originating from gas separation (dominated by hydrogen purification), the plant can achieve a positive NPV within four years of operation, driven by revenues from CO and low‐defect CNT products. This strategy ultimately provides a sustainable paradigm for simultaneous regeneration of deactivated catalysts and consumption of CO_2_, positioning microwave‐driven coke conversion as a promising platform for circular carbon economies.

## Conflicts of Interest

The authors declare no conflict of interest.

## Supporting information




**Supporting File**: advs73488‐sup‐0001‐SuppMat.docx.

**Supporting File**: advs73488‐sup‐0002‐Supplementary Video S1_Fe anchoring.mp4.

**Supporting File**: advs73488‐sup‐0003‐Supplementary Video S2_MAP‐on‐off.mp4.

## Data Availability

The data that support the findings of this study are available from the corresponding author upon reasonable request.
