# Supplementary material for: Microwave Ultrafast Heating on Iron‐based Nanogap Catalysts for CO2 Reduction Coupling with Coke Removal
Source: Adv Sci (Weinh). 2025 Dec 25;13(13):e18411. doi: 10.1002/advs.202518411 (PMC12955947; doi:10.1002/advs.202518411)
Supplement: Supplementary file 1 — Supporting File: advs73488‐sup‐0001‐SuppMat.docx. [file ADVS-13-e18411-s001.docx]

Supporting Information

**CO_2_ Reduction Coupling with Coke Removal from Iron-based Catalysts Achieved by Nanogap Induced Extreme Microwave Heating**

Xi Shen^a^, Zhenyu Zhao^a,*^, Jinsong Zhang^b^, Hong Li^a^, Xin Gao^a, c, *^

^a^ *School of Chemical Engineering and Technology, National Engineering Research Center of Distillation Technology, Collaborative Innovation Center of Chemical Science and Engineering (Tianjin), Tianjin University, Tianjin 300350, China*

*^b^ Institute of Structured and Architected Materials, Liaoning Academy of Materials, Shenyang 110167, China*

*^c^ Haihe Laboratory of Sustainable Chemical Transformations, Tianjin 300192, China*

Email: [zhaozhenyu2017@tju.edu.cn](mailto:zhaozhenyu2017@tju.edu.cn) (Zhao Z.) and [gaoxin@tju.edu.cn](mailto:gaoxin@tju.edu.cn) (Gao X.)

**1. Experimental Section for preparing coke model**

**1.1 Materials**

Iron(III) nitrate nonahydrate (Fe(NO_3_)_3_·9H_2_O) and citric acid were obtained from Tianjin Heowns Biochemical Technology Co., Ltd. Aluminum nitrate nonahydrate (Al(NO_3_)_3_·9H_2_O) was purchased from Shanghai Macklin Biochemical Technology Co., Ltd. High density polypropylene (PP) particles (4000 ± 500 mesh), with a softening point between 130 and 145 °C, were obtained from Shanghai Meryer Biochemical Technology Co., Ltd. Ferrocene was obtained from Shanghai Aladdin Biochemical Technology Co., Ltd. The nitrogen (N_2_) gas was purchased from Tianjin Liufang industrial gas distribution Co., Ltd. All reagents were of analytical grade and used as received without further purification.

1.2 Synthesis of catalysis for microwave pyrolysis

Preparation of FeAlOx precursors

Inspired by previous studies^[1-2]^, we adopted a flash Joule heating carbothermal reduction method to convert inexpensive precursors into nanogap catalysts (Fig. S1a). First, amorphous FeAlOx was prepared via a previously reported sol-gel method^[3]^. The FeAlOx precursors were prepared using a citric acid combustion method. Fe(NO_3_)_3_·9H_2_O and Al(NO_3_)_3_·9H_2_O were mixed in molar ratio of 1:2. The molar amount of citric acid was equal to Al(NO_3_)_3_·9H_2_O. Distilled water was added into the mixture to produce a gel, which was calcined in air at 350°C for 2 h. The obtained powder of FeAlOx were ground into fine particles for subsequent usage.

Thermal reduction treatment of precursors

Thermal shock of solid samples was achieved by electrical Joule heating in an argon-filled glovebox, as shown in Fig. S1. In this process, copper electrodes were connected by using a graphite paper, on which approximately 100 mg of the solid samples were placed. The external current source was supplied by a commercial thermal shock equipment (FJH-2024APLUS, Taiyuan, China) equipped with an IR thermometer.

| 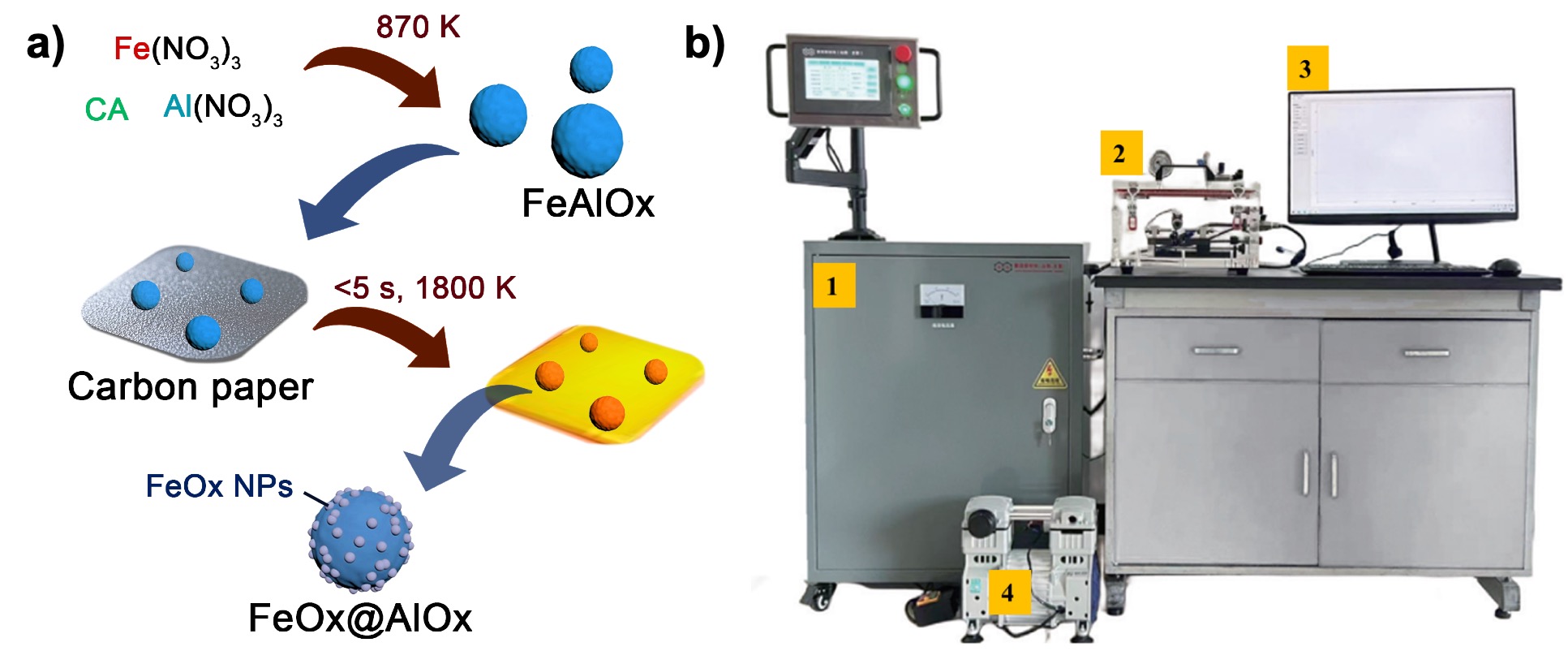 |
| --- |

Fig. S1 (a) Schematic illustration of catalyst preparation via the thermal shock method. (b) Photography of the used commercial equipment of thermal shock. 1-Power control cabinets; 2-Vacuum reaction chamber; 3-Signal acquisition equipment connected to a computer; 4-Vacuum pump.

The temperature sample was heated to 2300 K within 60 s and maintained for 300 s, during which the sample emitted strong light due to the blackbody irradiation (Fig. S2a). The time-varying temperature change during catalyst preparation is present in Fig. S2b. The reduction effect by carbon paper at elevated temperatures turned brown precursors into black powders (Fig. S2b).


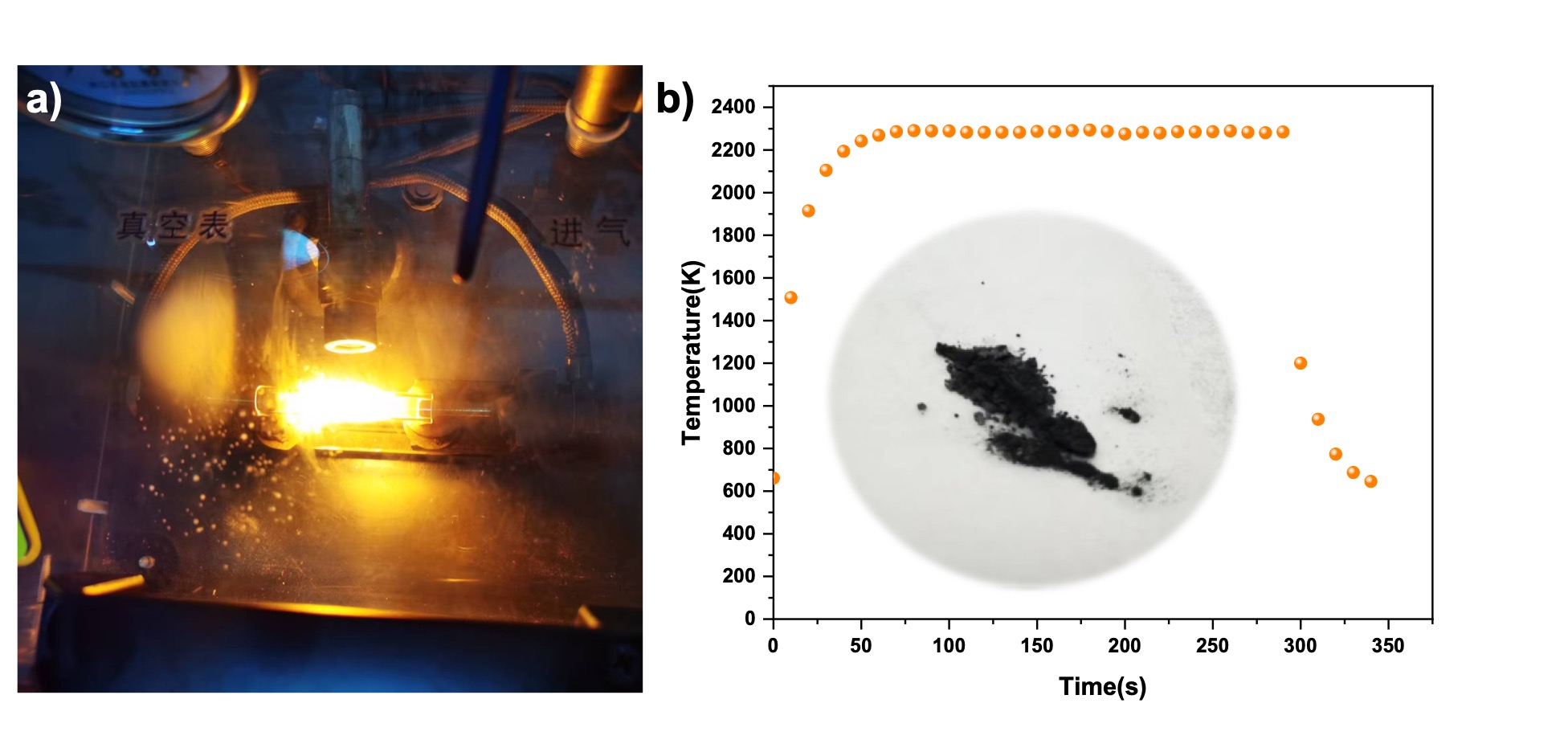


**Fig. S2** (a) Photography of luminescence during thermal shock of FeAlOx procedure. (b) Time-varying temperature change of the FeAlOx sample in the thermal shock equipment and photography of obtained FeOx@AlOx catalyst.

1.3 Microwave pyrolysis of PP

Experimental apparatus

The microwave heating equipment was purchased from MUEGGE Co., LTD. (Germany), consisting of a microwave head (MH015KS-314CN) equipped with a microwave power supply (MS015KE-111DE) with the maximal power of 15 kW, as demonstrated in Fig. S3. The generated microwave power was transmitted into a self-designed cavity (183.6 mm × 122.4 mm× 122.4 mm) through a rectangular waveguide (BJ22, 1090 mm× 550 mm). The unabsorbed microwave energy was reflected into a circulator (MW1001B-210DC) and finally absorbed by a water load (MW1021E-230DF).


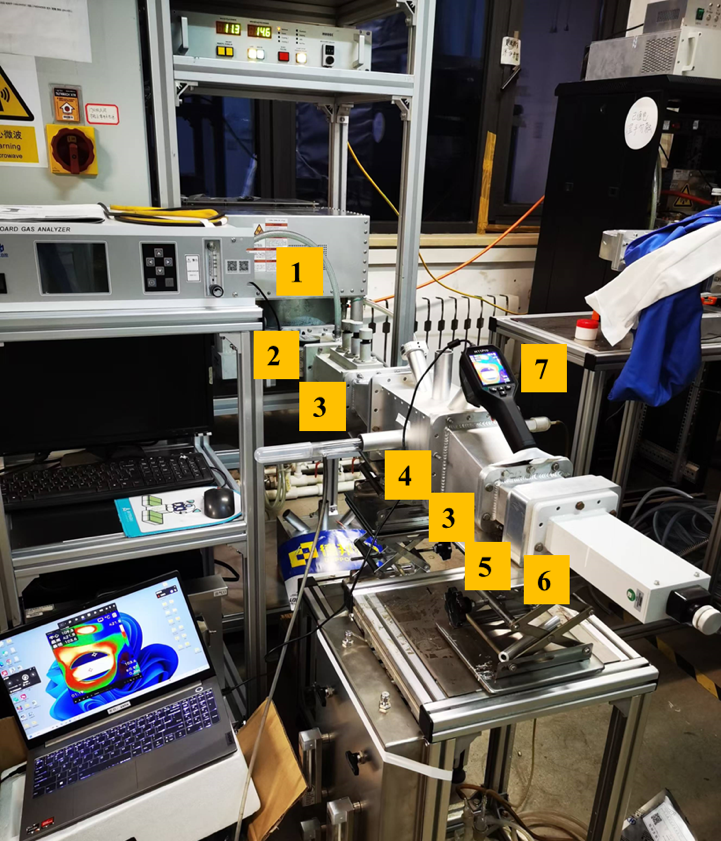


**Fig. S3** Photography of experimental apparatus for conducting microwave shock pyrolysis of plastics. 1-microwave generator; 2- circulator and microwave reflection measurement instrument; 3-Transitional waveguides; 4-microwave cavity; 5-rectangular waveguide; 6-adjustable short-circuit plunger; 7-IR camera.

Experimental procedure of microwave assisted pyrolysis

A quartz boat containing the mixture of plastic and prepared catalysts was located in the middle a quartz tube reactor (with the inner diameter of 20 mm). Nitrogen with the flow rate maintaining at 200 mL/min flows the tube reactor to act as protective gas. The tube reactor pass through the microwave cavity, where the sample was irradiated by microwave. The cavity is connected to a microwave generator (maximal power is 15 kW) via a BJ22 waveguide, through which microwave energy is transmitted to the sample. The cavity has three cylindrical windows for a fiber spectrometer (Ocean Optics, USB 2000+), an infrared thermal camera (Hikvision, HM-TD2037T-4/X) and a camera (Huawei, Leica shot) to monitor the phenomenon during microwave heating. During microwave irradiation, the camera was utilized to continuously record the discharge phenomenon of the sample, whose luminescence was monitored by the fiber spectrometer. Surface temperature values of the tube reactor was measured by the infrared thermal camera. Besides, the gas products from plastic pyrolysis were collected by using gas analyzer (Gasboard-3100, Ruiyi Co. Ltd., China) while the generated solid residues were collected for structure/composition characterization and further usage.

1.4 Characterization of solid residues after microwave pyrolysis

Characterization methods for solid residues in this study

The elemental content was measured by using Inductively coupled plasma-mass spectroscopy (ICP-MS) analysis (Agilent 5110), where the solutions were prepared by digesting the catalysts in aqua regia follower by dilution with 2% hydrochloric acid. The hydrogen content of gas products was measured by using a gas chromatography (Haixin-GC-950) equipped with carbon molecular sieve packed column (3 m, 80-100 mesh) and a TCD detector while the species of each peak was determined by using a standard gas bag, where Ar (99.99%) was used as carrier gas. The temperature values of gasification chamber, column oven and detector were determined at 120°C, 100°C and 120°C, respectively. The generated thimbleful liquid attached on the inner wall of tube reactor after several cycles was diluted by using octane and characterized by using a GC-MS (Shimadzu GCMS-QP2020NX). X-ray diffraction (XRD) patterns of catalyst particles and solid residuals were obtained by a Japan Rigaku SmartLab SE X-ray diffractometer equipped with a Cu Kα radiation source (λ=1.5418 A), where the voltage and current were set at 40 kV and 40 mA, respectively. The microstructure and morphology of solid products were characterized by using scanning electron microscopy (SEM, TESCAN MIRA LMS at 15kV). The solid products powders were dispersed in ethanol solvent and treated by ultrasonic method for 5 minutes. The samples were dripped onto conductive adhesive, dried and sprayed with gold for 45 seconds at 10 mA using Quorum SC7620 sputtering coating machine. EDS linear scanning and EDS mapping were obtained by setting the accelerated voltage at 15kV.

Morphology characterization of solid residues after microwave pyrolysis

SEM imaging (Fig. S4a) showed the solid residues after five-cycle microwave pyrolysis of PP are mainly composed of carbon nanotubes (CNTs) with average diameters of 29 nm, while TEM (Fig. S4b) confirmed uniform dispersion of Fe nanoparticles (10–20 nm) within CNTs, consistent with the “solution-diffusion-recrystallization” growth mechanism^[4]^.


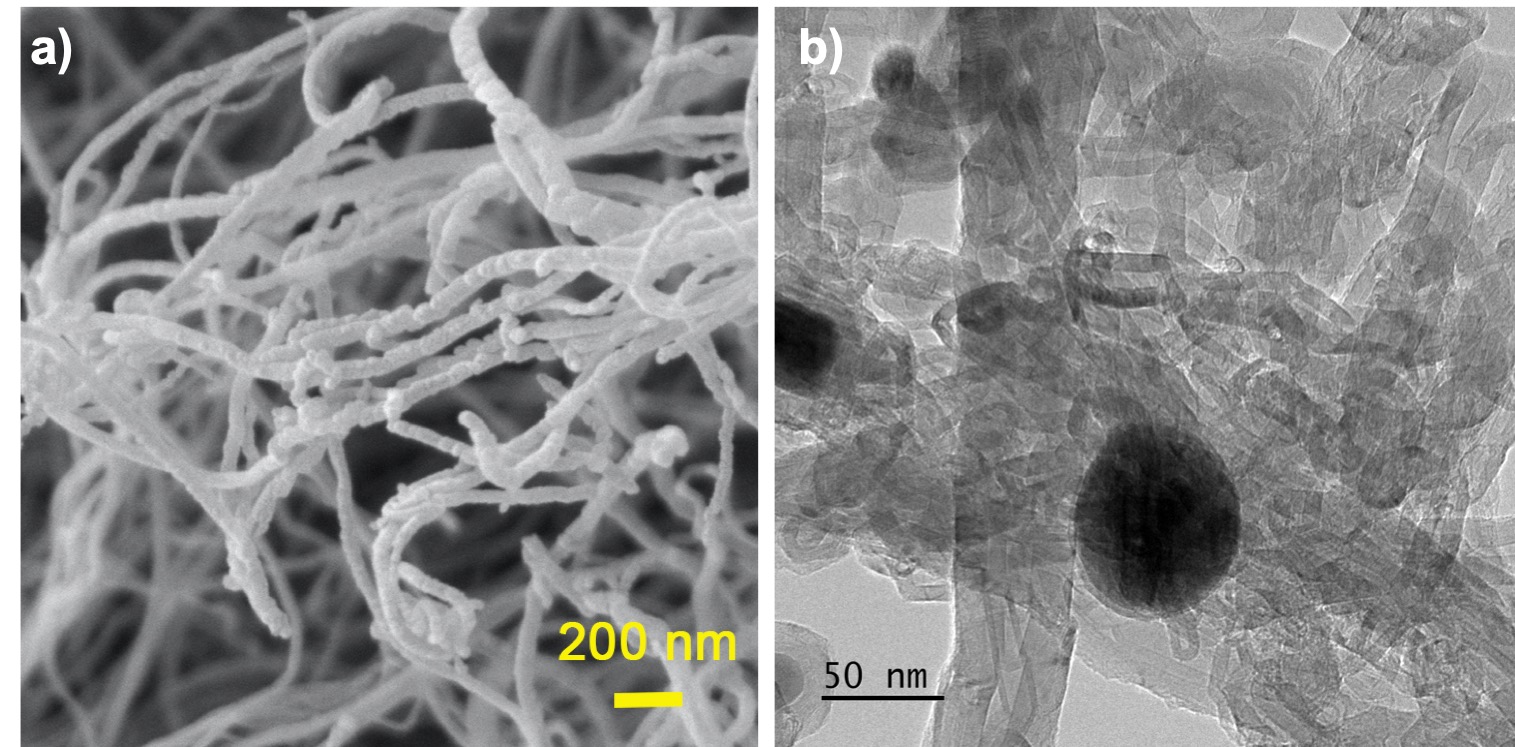


**Fig. S4** (a) SEM imaging and (b) TEM imaging of solid residues after five-cycle microwave pyrolysis of PP.

**2. Experimental Section for microwave induced iron deposition**

2.1 Measurement and calculation of ultra-high temperatures

The cavity has three cylindrical windows for a fiber spectrometer (Ocean Optics, USB 2000+), an infrared thermal camera (Hikvision, HM-TD2037T-4/X) and a camera (Huawei, Leica shot) to monitor the phenomenon during microwave heating. During microwave irradiation, the camera was utilized to continuously record the discharge phenomenon of the sample, whose luminescence was monitored by the fiber spectrometer. Surface temperature values of the tube reactor was measured by the infrared thermal camera. The spectra of emitted light from the irradiated sample are recorded by a fiber spectrometer (Ocean Optics, USB 2000+). The luminescence intensity (I) of the sample’s spectra were nonlinearly fitted based on Planck’s blackbody radiation formula, as exhibited in Eq. (1).

|  | $I(\lambda,T)=\frac{8\pi h}{\lambda^{3}}\frac{1}{e^{{hc}/{\lambda kT}}-1}$ | (1) |
| --- | --- | --- |

where $\pi$, *h*, *c* and *k* represent circular constant, Planck constant, light velocity and Boltzmann constant, respectively. By fitting the wavelength (λ)-varying luminescence spectra, the temperature of sample (T) can be quantitatively determined.

Here a non-contact colorimetric thermometry approach based on the Planck blackbody radiation law was employed to confirm the accuracy of spectra-fitting method for temperature measurement of samples irradiated by microwave. According to Planck’s blackbody radiation theory, the thermal radiation intensity of a substrate at specific wavelength is only dependent on its temperature. In principle, the thermal radiation spectra measured during microwave heating should be consistent with that obtained under conventional heating. As depicted in Fig. S5a, there is a strong emissive peak in the range of 550-1050 nm in the samples. The intensity of luminescence has a significant rise with the increase in microwave power. Considering the solid residue of pyrolysis is not exactly black-body source, a modified gray-body equation is used to fit the luminescent spectra to determine the localized temperature of samples. It should be noted that there exists an emission drop at nearly 890 nm, which can be ascribed to the absorption of infrared light by SiO_2_ in quartz reactor wall. Here the luminescent spectra data in the range of 550 nm-800 nm are used for roughly estimating the sample temperature. Here the luminescent spectra of samples at various temperature points in a commercial thermal shock equipment are monitored during thermal shock treatment of FeAlOx. The schematic representation of samples in Fig. 5b showed the change of luminescent color from red to orange with the increase of heating temperature; this blue shift behavior leads to the increase of thermal radiation intensity ratio (I_830 nm_ / I_900 nm_). Therefore, a standard curve of thermal radiation intensity ratio (I_830 nm_ / I_900 nm_) versus temperature is obtained as shown in Fig. 5b, which can be used to quantitatively calculate the sample temperature, which agrees well the fitted values. The experimental results confirmed that the spectra-fitting method for obtaining actual sample temperatures.


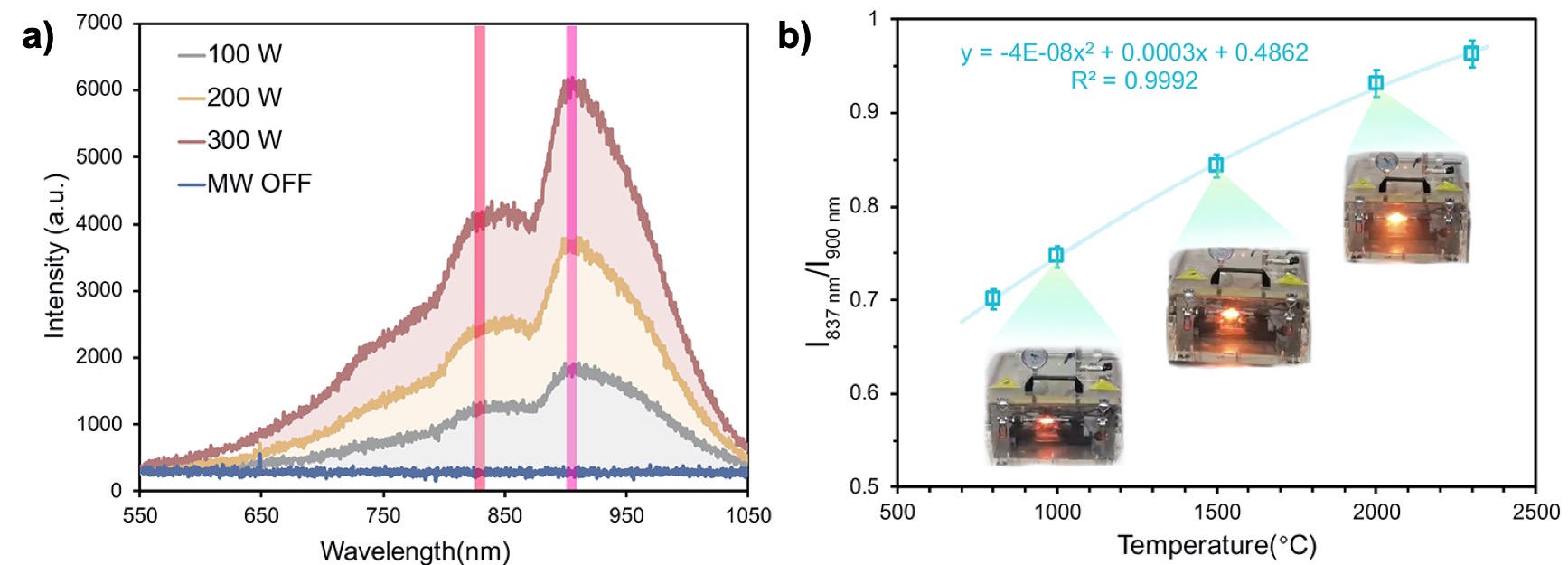


**Fig. S5** (a) IR-vis spectra of emission from the sample heated by microwave. (b) Standard curve between temperature and luminescent intensity ratio I_837 nm_ / I_900 nm_.

2.2 Structural characterization of iron anchored CNTs

ICP-MS analysis to determine the sample’s iron content


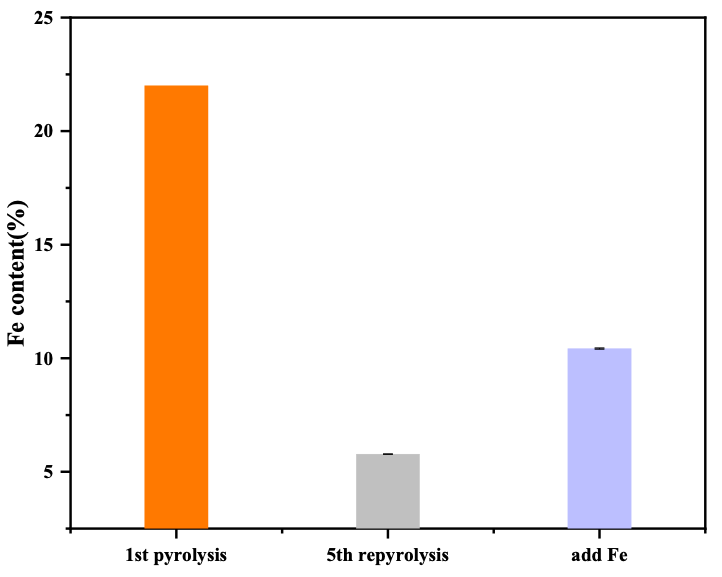


**Fig. S6** ICP-MS analysis of solid products obtained after 1st cycle (1^st^ pyrolysis) and 5th cycle (5^th^ pyrolysis) of microwave assisted pyrolysis (MAP) and the coke sample anchored by iron nanoparticles (add Fe).

XRD analysis to determine the sample’s crystalline structure


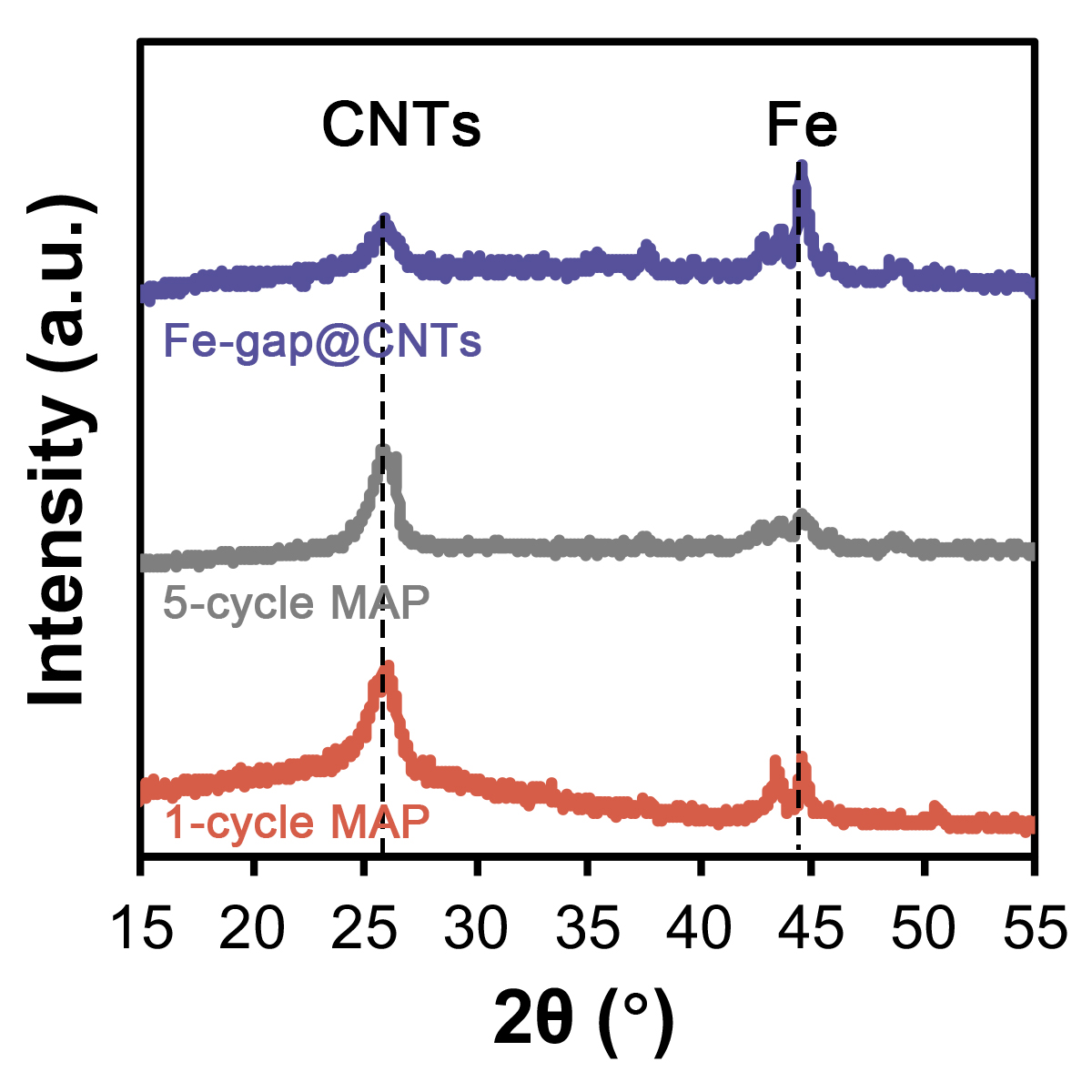


**Fig. S7** XRD patterns of solid products obtained after 1st cycle and 5th cycle of microwave assisted pyrolysis (MAP) and the coke sample anchored by iron nanoparticles (Fe-gap@CNTs).

STEM analysis to determine the size and its distribution of iron nanoparticles


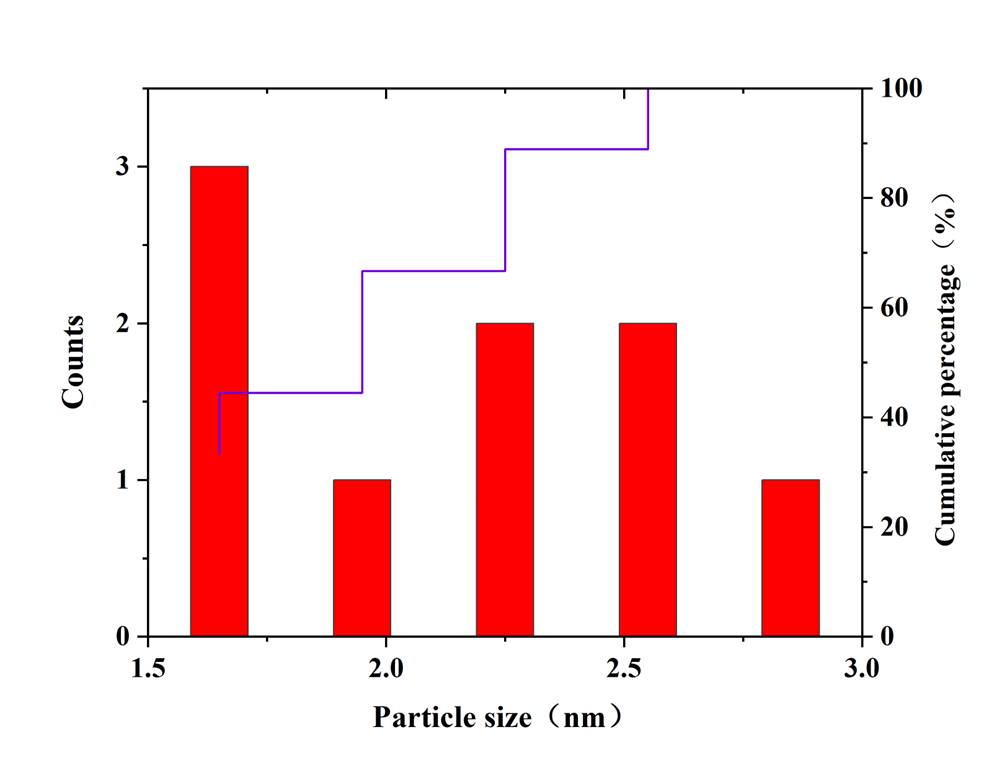


**Fig. S8** Size distribution of nanoparticles anchored on the CNTs after microwave induced iron deposition.


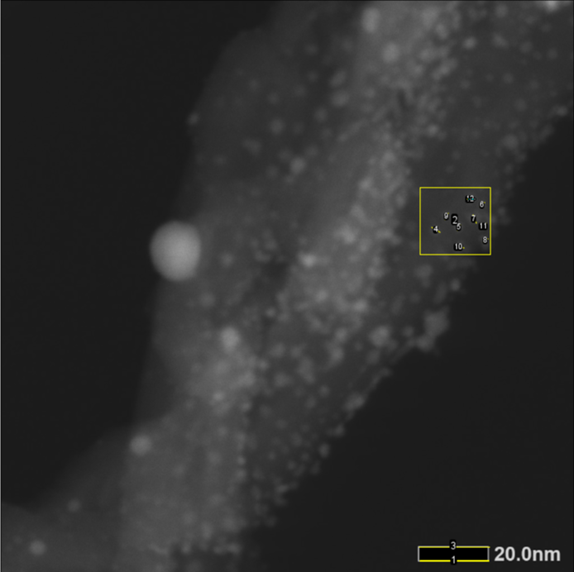


**Fig. S9** TEM image of Fe-gap@CNTs. Within a square box with a side length of 20 nm, at least 12 visible iron nanoparticles can be counted.

**3. Experimental Section for microwave induced coke elimination**

3.1 Observation of experimental phenomenon during microwave irradiation

Luminescence monitoring to determine the performance of equipment operating


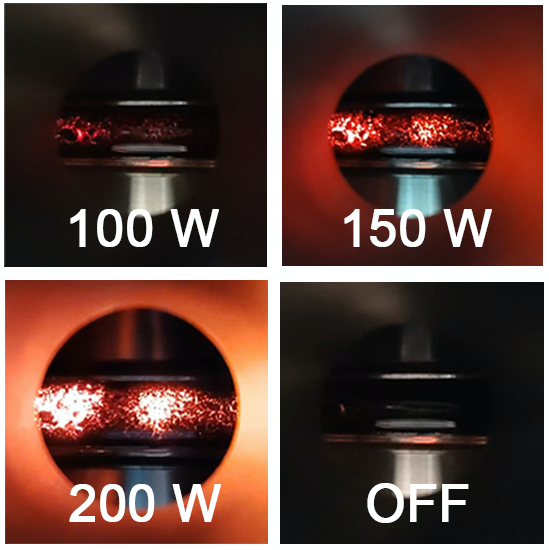


**Fig. S10** Images of luminescence from the sample irradiated by microwave with various power.

3.2 Characterization of solid residues after coke elimination

Mass change measurement to determine the quantity of removed carbon


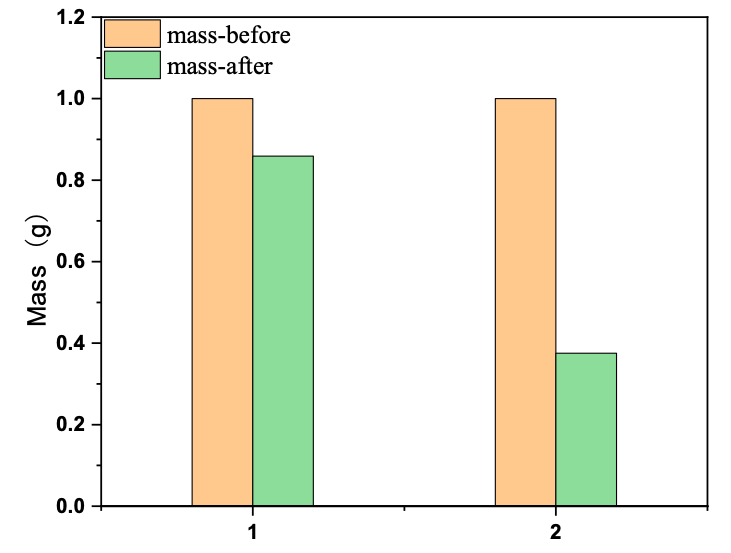


**Fig. S11** Mass weight of the sample before and after microwave induced coke elimination in CO_2_ atmosphere. 1-CNTs without iron anchoring irradiated by 200 W microwave for 30 min, 2- Fe-gap@CNT irradiated by 200 W microwave for 30 min.

3.3 Temperature distribution of reactor wall monitored by IR camera


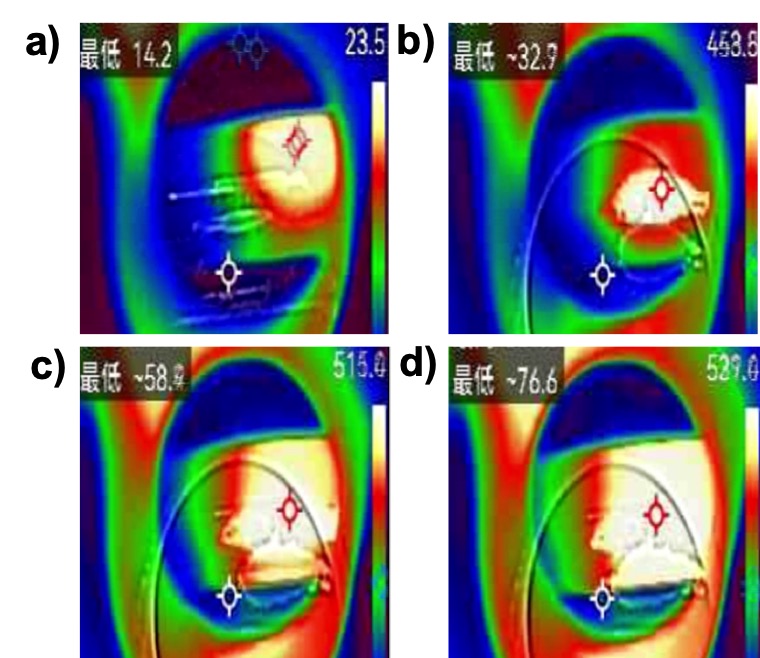


**Fig. S12** Reactor wall during microwave induced coke of PP monitored by IR camera at (a) 0.1 min, (b) 4 min, (c) 6 min, (d) 8 min after turning on the microwave generator.


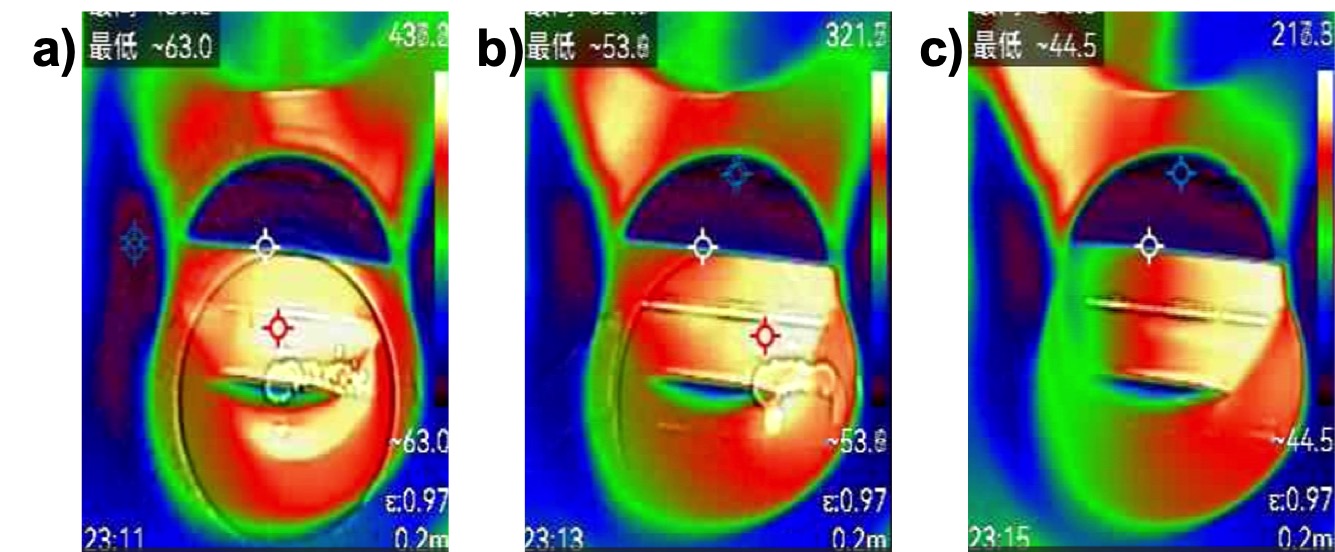


**Fig. S13** Reactor wall during microwave induced coke of PP monitored by IR camera at (a) 0.5 min, (b) 1.5 min, (c) 2.5 min after turning off the microwave generator.

**4. Numerical simulation for thermal radiation during microwave heating**

4.1 Physical model

We establish a physical model of tube reactor in in COMSOL Multiphysics V6.3 to simulate the temperature (T) change of the tube reactor wall heated by the thermal radiation of discharging samples, as shown in Fig. S14. A circular tube with the diameter of 20 mm is built to represent the quartz tube reactor used in microwave pyrolysis experiments. A disc-shape object with the diameter of 10 mm is located in the middle of the quartz tube reactor to represent the sample irradiated by microwave.

|  |
| --- |

Fig. S14 Physical model of numerical simulation for thermal radiation.

4.2 Control equations

The numerical simulation is conducted under the assumption that the physical system mainly includes the thermal radiation and conductive cooling processes, which are controlled by the following Equations (Eq. (2) and Eq. (3)).

|  | $\rho_{t}C_{p,t}\frac{\partial T}{\partial t}=\nabla\cdot\left( k_{t}\nabla T \right)+Q_{s}$ | (2) |
| --- | --- | --- |
|  | $-n\cdot(-k_{g}\nabla T)=h(T_{inf}-T)+\frac{\varepsilon}{1-\varepsilon}(J_{0}-\sigma T^{4})$ | (3) |

where $\rho_{t}$ $C_{p,t}$ and $k_{t}$ represent the density, thermal capacity and thermal conductivity of the tube reactor. Qs represents a volumetric heating source term, which can be calculated according to Eq. (3), in which n is the surface normal vector. T_inf_ and k_g_ represent the temperature of convective cooling gas outside the reactor and the thermal conductivity of flowing gas through the reactor, respectively. $\varepsilon$, *J*_0_ and $\sigma$ represent emissivity, radiant intensity and Stefan-Boltzmann constant.

In the simulation, the discharging sample is regarded as an entity object, whose radiation power is mainly determined by its localized temperature. The thermal energy launched from the sample is absorbed by the reactor wall, inducing its temperature rise.

To conduct numerical simulation, we use free triangular meshes with general size for discretization of the established model while triangular meshing subdivision is employed for the sample, as shown in Fig. S4.

| 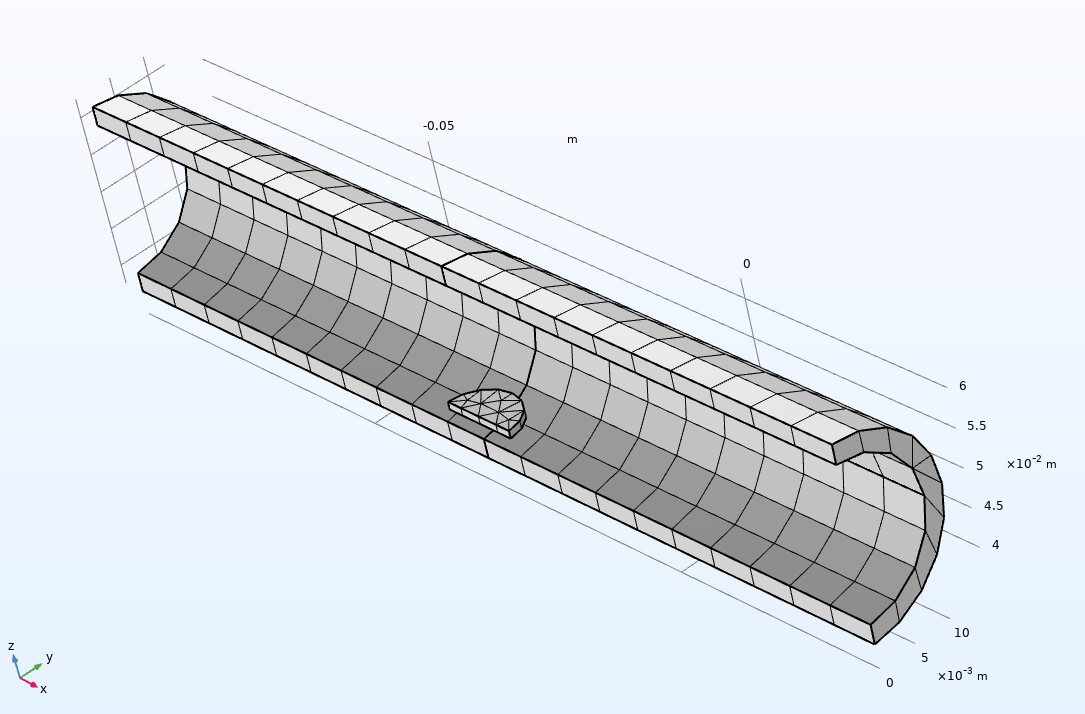 |
| --- |

Fig. S15 Meshes used for conducting numerical simulation.

4.3 Key parameters

The physical properties of sample and reactor tube are listed as Table S1.

Table S1 Physical properties of materials

| Properties | Discharging sample | Reactor wall |
| --- | --- | --- |
| *k* (W·m^-1^·K^-1^) | 400 | 163 |
| *ρ* (kg·m^-3^) | 8700 | 2330 |
| *Cp* (J·kg^-1^·K^-1^) | 10 | 703 |
| $\varepsilon$ | 0.99 | 0.5 |

Before the analysis of thermal radiation process, the cooling process of reactor tube in the absence of thermal radiation source is simulated for determining the key parameter (the heat transfer coefficient of gas, *h*_gas_) to ensure the accuracy of numerical simulation. In this case, the initial temperature of reactor wall is set to be 730 K (the measured value in experiments). Then the temperature decrease curves at Point A with various *h_gas_* are calculated as shown in Fig. S16.


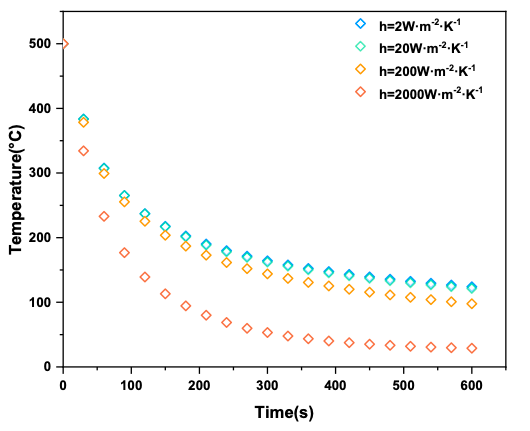


Fig. S16 Cooling process of reactor wall simulated with different *h_gas_* value.

It can be concluded that the temperature change simulated in COMSOL Multiphysics when the value of *h_gas_* is equal to 20 W·m^-2^·K^-1^ agrees well with the temperature measured by IR camera in experiments (Fig. S13).

The initial temperature of reactor wall is set to be 298 K and the temperature rise at Point A in Fig. S7 is monitored during thermal radiation. The parameters for conducting numerical simulation are listed in Table S2.

Table S2 Parameters for conducting numerical simulation

| Parameters | Value [Unit] | Description |
| --- | --- | --- |
| *T_wall_* | 298 K | Initial temperature of reactor wall |
| *T_gas_* | 298 K | Gas temperature |
| *h_gas_* | 20 W·m^-2^·K^-1^ | Heat transfer coefficient of gas |

4.4 Simulation results

The time-varying temperature distribution of reactor wall is obtained by using transient solver with the step size of 10 seconds. The thermal radiation heating under various radiation power is calculated is calculated. For instance, the thermal distribution of the tube reactor after 10 minutes under 58.6 W/cm^2^ is demonstrated in Fig. S17.

|  |
| --- |

Fig. S17 Temperature distribution of reactor wall calculated under 58.6 W/cm2 at different time: (a) 30 s, (b) 60 s, (c) 300 s.

Besides, the temperature change at Point A is monitored under various power of thermal radiation, as exhibited in Fig. S18.


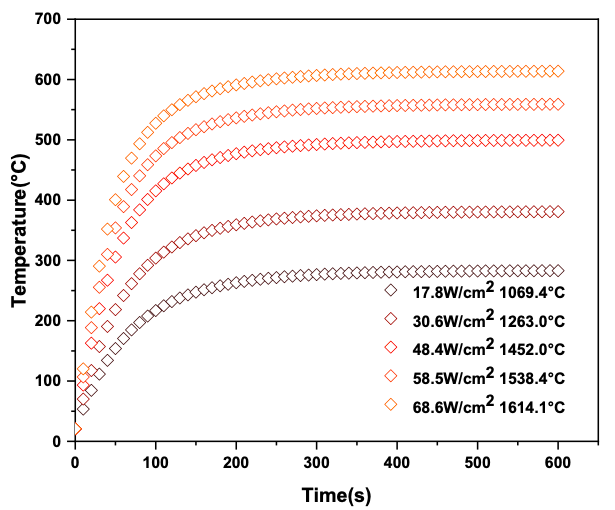


Fig. S18 Time-varying temperature change at Point A under thermal radiation with various power.

**5. Numerical simulation for nanogap concentrated electromagnetic field**

5.1 Physical model

We establish a physical model of nanoparticles decorated surface in COMSOL Multiphysics V6.3 to simulate the distribution of microwave field near iron nanoparticles anchored on carbon nanotubes.

The geometry consists of a semi-infinite dielectric substrate (representing the coke surface) occupying the half-space z<0 and nitrogen gas (z>0), with several iron nanoparticles positioned at the substrate-air interface, as shown in Fig. S19.


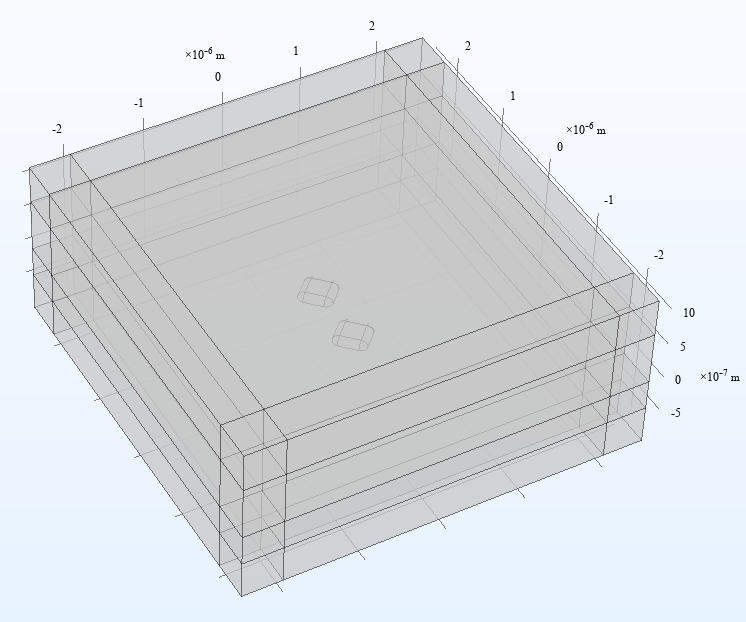


**Fig. S19** Physical model of numerical simulation for nanogap concentrated electromagnetic field.

5.2 Control equations

A TE plane wave with a wavelength of centimeters is incident at polar angle θ and azimuthal angle ϕ, with its electric field vector polarized along the ϕ-direction (orthogonal to the plane of incidence), to represent the microwave irradiation. The simulation employs the Electromagnetic Waves, Frequency Domain interface to solve Maxwell’s equations in the frequency domain. For a time-harmonic field with angular frequency ω (= 2π*f*, where *f* is the frequency of electromagnetic field), the governing equation for the electric field E is shown in Eq. (4)

|  | $\nabla\times\left( \frac{1}{\mu_{r}}\nabla\times\boldsymbol{E} \right)-k_{0}^{2}\varepsilon_{r}\boldsymbol{E}=0$ | (4) |
| --- | --- | --- |

where *μ*_r_ is the relative permeability, *ε*_r_ is the relative permittivity, and *k*_0​_ =2π/λ ( λ is the free-space wavelength.

In the simulation, Periodic Structure Node was applied to model the infinite extension of the substrate in the xy-plane. It automatically configures Periodic Ports that defines the incident wave (input port) and absorb the transmitted wave (output port). Besides, Floquet Periodic Conditions enforces periodicity with a complex phase factor, ensuring the field on one boundary equals the field on the opposite boundary multiplied by exp(−*i*(*k*_x_​x+*ky*y)), where k_x_ and k_y_ are the in-plane wave vector components. Besides, Perfectly Matched Layers (PMLs) are used to surround the computational domain to absorb scattered fields without reflection, minimizing artificial boundary effects. The above setup enables accurate computation of how the nanoparticle modifies the incident electromagnetic field.

To conduct numerical simulation, we use free triangular meshes with general size for discretization of the established model while triangular meshing subdivision is employed for the sample, as shown in Fig. S20.


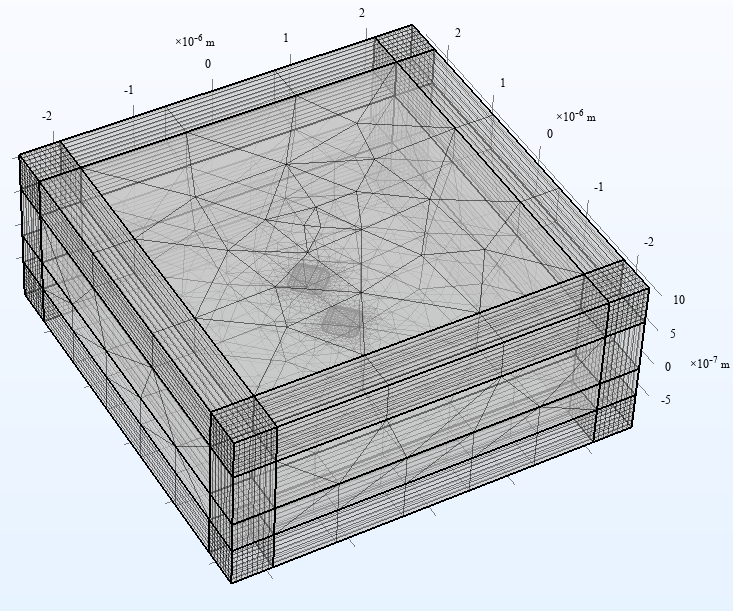


Fig. S20 Meshes used for conducting numerical simulation.

5.3 Key parameters

The physical properties of sample and reactor tube are listed as Table S3.

Table S3 Physical properties of materials

| Properties | Value | Definition |
| --- | --- | --- |
| *h_air_* (nm) | 700 | Air domain height |
| *h_subs_* (nm) | 400 | Substrate domain height |
| *I_0_* (MW/m^2^) | 1 | Intensity of electromagnetic field |
| φ | 0 | Azimuthal angle |

5.4 Simulation results

First, the size of iron nanoparticles is set to be 400 nm, while the gap distance between the two nanoparticles is set to be 200 nm. The simulating intensity distribution electromagnetic field is demonstrated in Fig. S21.


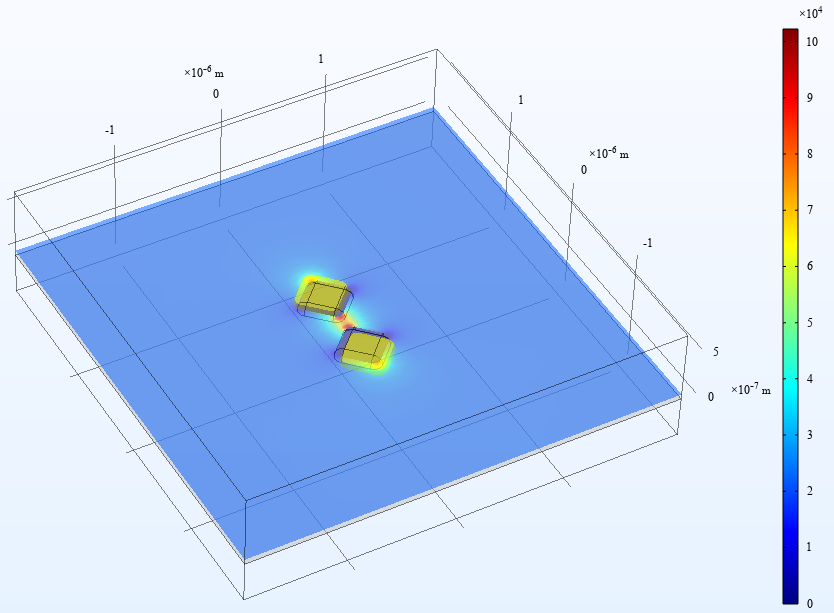


**Fig. S21** Simulated intensity distribution of electromagnetic field intensity between nanoparticles.

Subsequently, we conducted a parametric scanning to investigate the influence of iron content and gap distance on the concentration effect of electromagnetic field. The simulation results are demonstrated in Table S4.

**Table S4** Simulating results of parametric sweep analysis

| Gap distance (nm) | Iron content | E_max_ (V/m) |
| --- | --- | --- |
| 60 | 0.64 | 66380 |
| 60 | 0.81 | 68786 |
| 60 | 1 | 73444 |
| 60 | 1.21 | 77012 |
| 60 | 1.44 | 81332 |
| 100 | 0.64 | 49254 |
| 100 | 0.81 | 53095 |
| 100 | 1 | 56400 |
| 100 | 1.21 | 58358 |
| 100 | 1.44 | 62139 |
| 140 | 0.64 | 42425 |
| 140 | 0.81 | 45120 |
| 140 | 1 | 47235 |
| 140 | 1.21 | 49574 |
| 140 | 1.44 | 51884 |
| 180 | 0.64 | 37021 |
| 180 | 0.81 | 39244 |
| 180 | 1 | 41456 |
| 180 | 1.21 | 43575 |
| 180 | 1.44 | 45772 |
| 220 | 0.64 | 33530 |
| 220 | 0.81 | 35469 |
| 220 | 1 | 37526 |
| 220 | 1.21 | 38991 |
| 220 | 1.44 | 40585 |

**6. Structural characterization of solid residues**

6.1 Characterization of catalyst structure in microwave pyrolysis

Morphology change of FeAlOx catalyst after thermal shock treatment


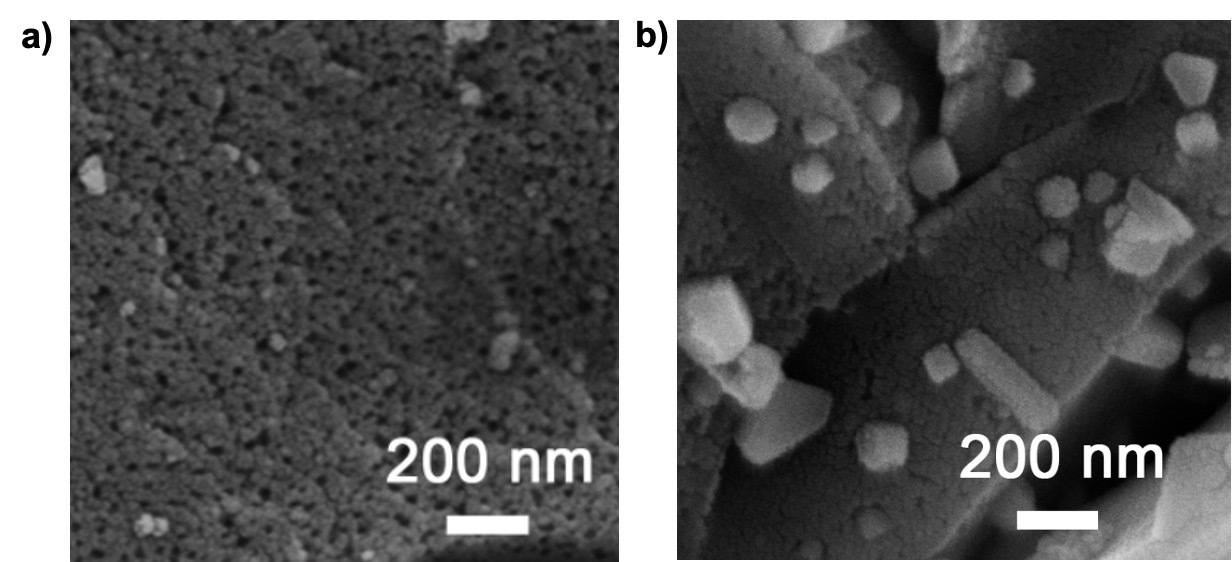


**Fig. S22** SEM images of FeAlOx catalyst before (a) and after (b) thermal shock treatment at 2300 K.

Crystalline structure change of FeAlOx catalyst after thermal shock treatment


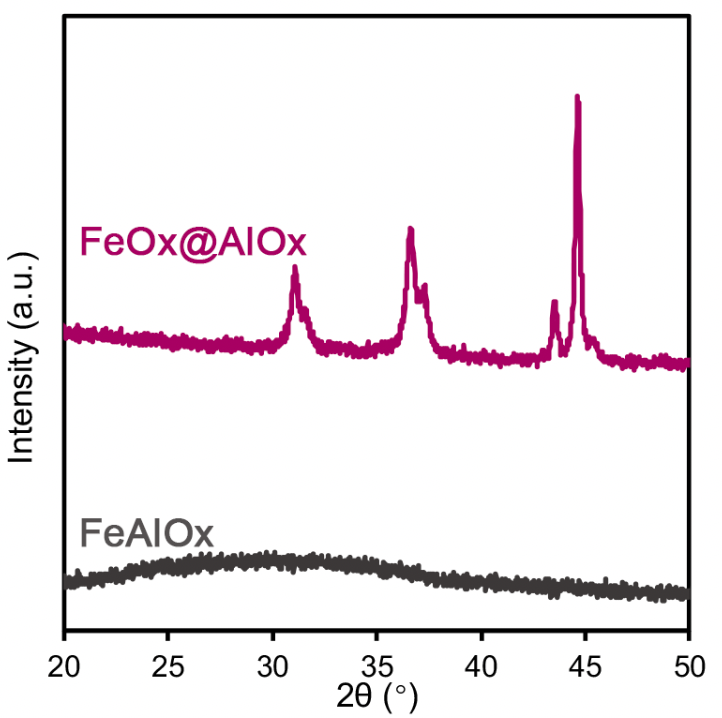


**Fig. S23** XRD patterns of FeAlOx catalyst before and after (entitled as FeOx@AlOx) thermal shock treatment at 2300 K.

6.2 Comparison of microwave absorbing capability

We used the established equipment to conduct the heating rate test under microwave irradiation on the catalysts that have been previously reported in the previous study, as shown in Fig. S24. The blue columns represent the results reported in the literature^[5-10]^, while the red columns are for the catalysts tested in this paper. Generally speaking, there are roughly three methods for the preparation of microwave-responsive materials. The first type (I) of catalyst is prepared by coating the catalyst onto the surface of microwave-absorbing carriers. The second type is based on the size effect (II), that is, by adjusting the microscopic size of the catalyst to regulate the microwave-absorbing properties of the material. The third type (III) is the one reported in this paper, which involves arranging iron nanoparticles to form nanogaps so as to enhance the local electromagnetic field intensity.

Fe/SiO_2_, Fe/SiC and Fe/Carbon represent the loading of iron nanoparticles on various support, where their heating rates are mainly determined by the dielectric loss of the support. FeAlOx-2000 represents the FeAlOx treated with 2000°C thermal shock treatment. The increase of treatment temperature enlarges their microwave heating rates, which can be mainly attributed to the formation of iron-containing nanoparticles (characterized by Fig. S22 and Fig. 23). Compared to FeAlOx-2000, Fe-gap@CNT has significantly larger number density of nanoparticles and narrower spacing between particles, allowing this structure to be rapidly heated, with a ramp rate of 11057 K/min.


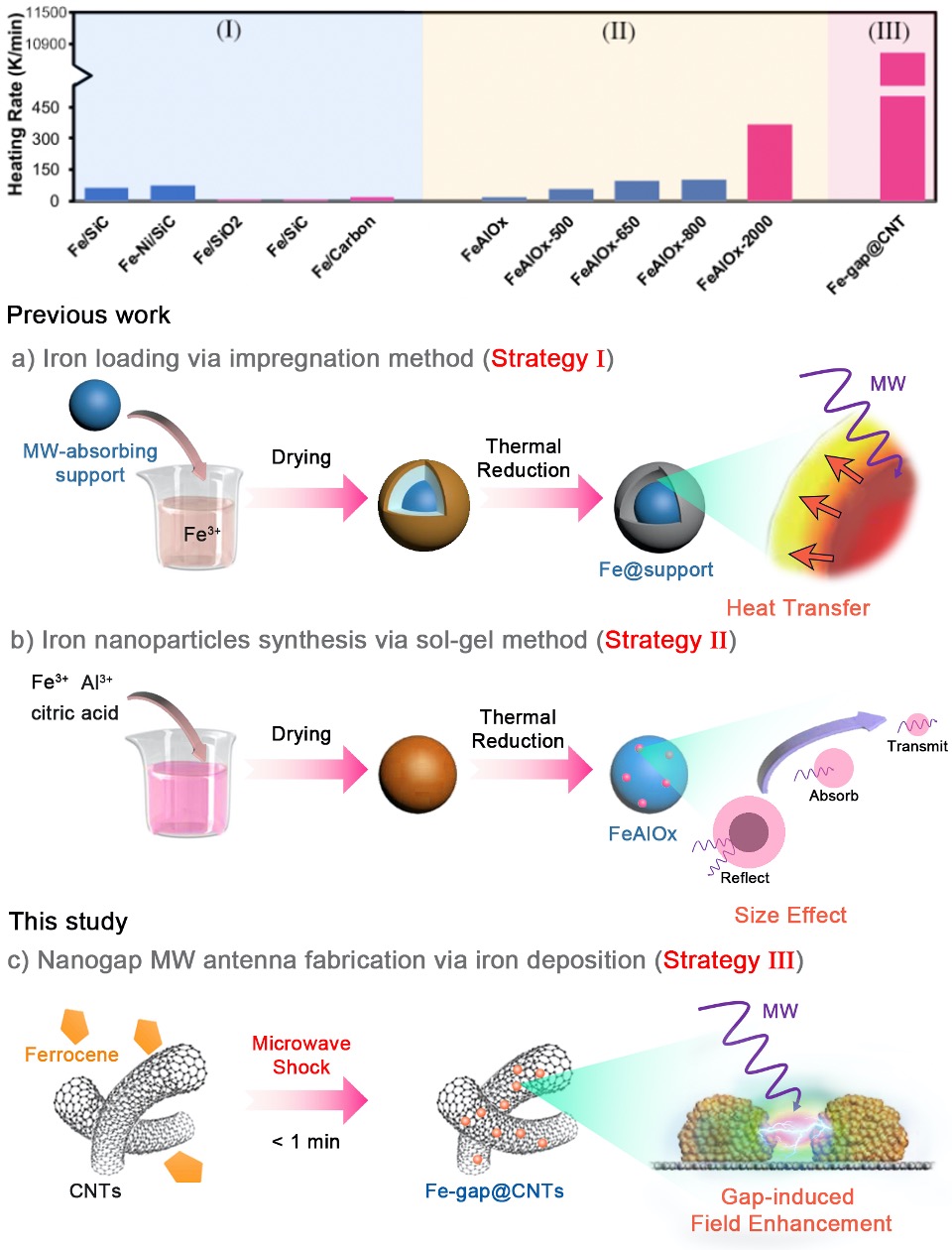


**Fig. S24** Microwave heating rates comparison of microwave-responsive materials.


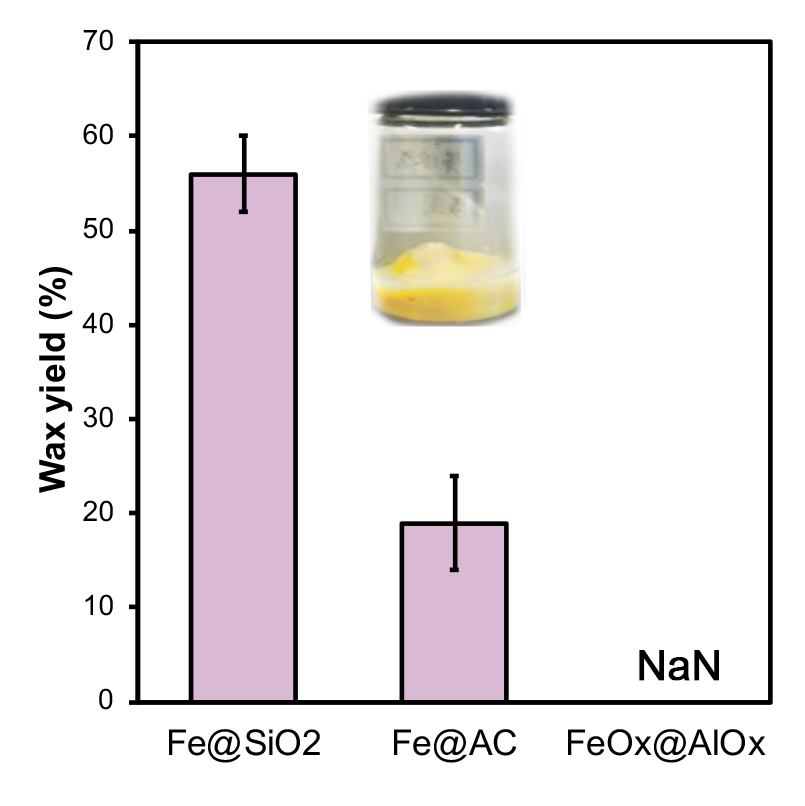


**Fig. S25** The yield of wax during microwave assisted pyrolysis by using different catalysts.

6.3 Structural change of iron catalyst during multiple cycle pyrolysis


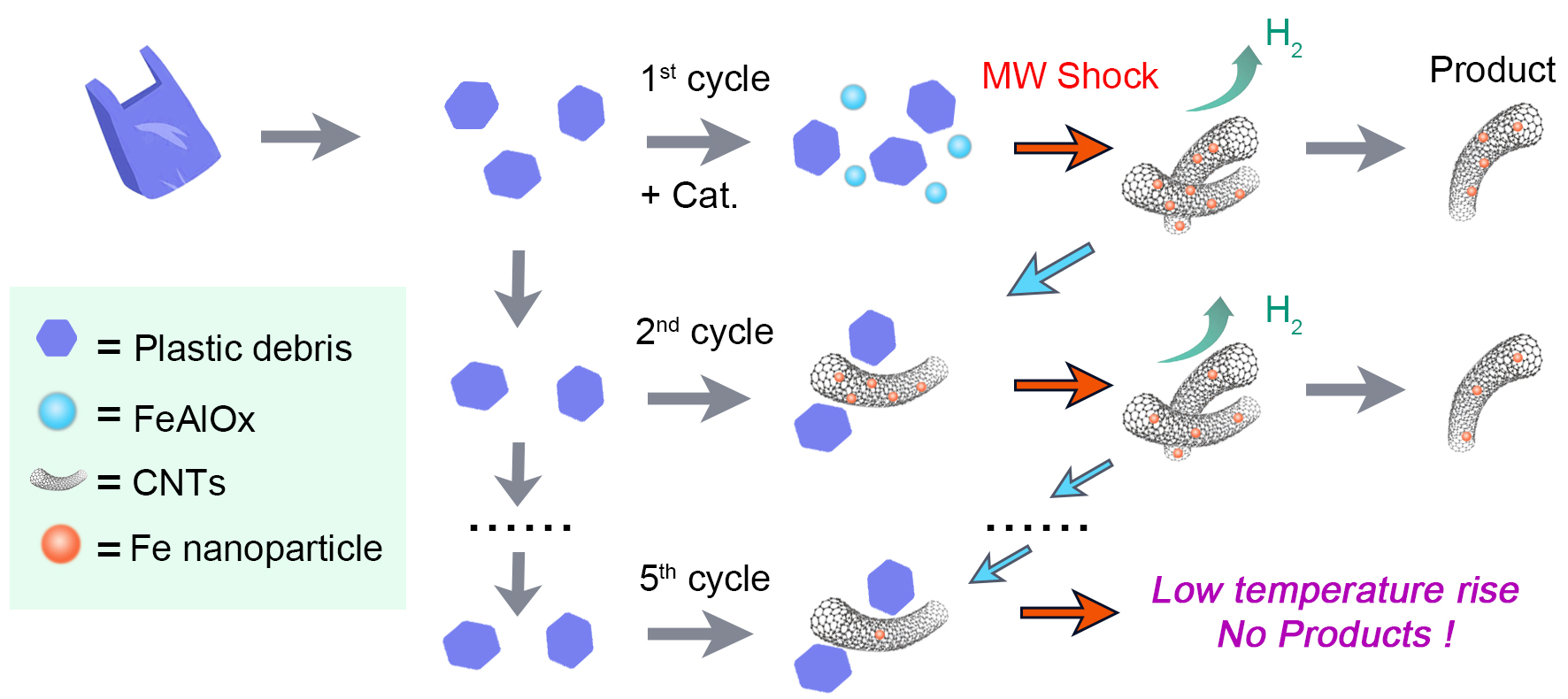


**Fig. S26** Schematic illustration of microwave pyrolysis of PP.

**Fig. S27** (a) Schematic illustration of tip-growth behavior of CNTs and (b) the resulting iron particle migration and separation of Fe from the Al-O framework, evidenced by SEM linear scanning analysis.

**Fig. S28** Temperature increasing rate of reactor wall during multiple cycle of microwave pyrolysis caused by the thermal radiation of ultrahigh-temperature sample irradiated by microwave.

6.4 Performance of CNTs after coke elimination

X-ray photoelectron spectroscopy (XPS) analysis indicates that the C 1s spectrum of CNTs obtained from PP pyrolysis and CO_2_ reforming is dominated by a sharp peak (Figure S28(b)) corresponding to sp^2^-hybridized carbon. Similar with commercial CNTs (Figure S28(a)), the signals for sp^3^ carbon and C-O bonds are almost negligible, demonstrating the high chemical purity and the near-perfect graphitic structure of the carbon, with minimal presence of undesirable functional groups or structural imperfections.


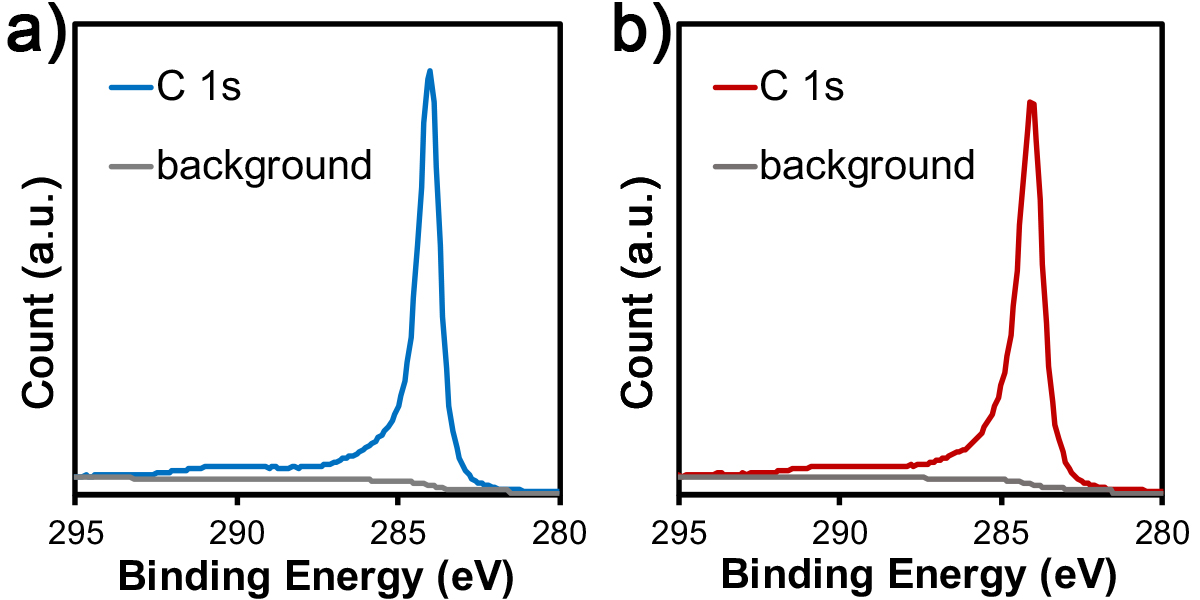


**Fig. S29** XPS results of (a) commercial CNTs and (b) CNTs obtained from PP pyrolysis and after coke removal (CO_2_ reforming)

Temperature-programmed oxidation (TPO) analysis was performed by following the below procedures. A 100 mg sample was weighed and transferred to a reaction tube. The sample was pre-dried via programmed heating from room temperature to 120 °C at a rate of 10 °C/min, followed by purging with a helium (He) flow (50 mL/min) for 1 h. After cooling to 50 °C, desorption was performed by heating to 900 °C at 10 °C/min under a 10% O₂/He gas mixture. The desorbed gases were detected using a thermal conductivity detector (TCD), and mass spectrometry targeted the following mass-to-charge ratios (m/z): H₂ (2), CO (28), and CO₂ (44), as shown in Figure S29(a). The test results are demonstrated in Figure S29 (b-d). The elimination of these unmanageable defects generated during PP pyrolysis enables the production of near-perfect CNTs, showing a high onset temperature for oxidation than Fe-anchored commercial CNTs (Figure S29). These high-quality CNTs can serve as cost-efficient raw materials of defect engineering^[11]^ for nanoelectronics and energy storage applications.


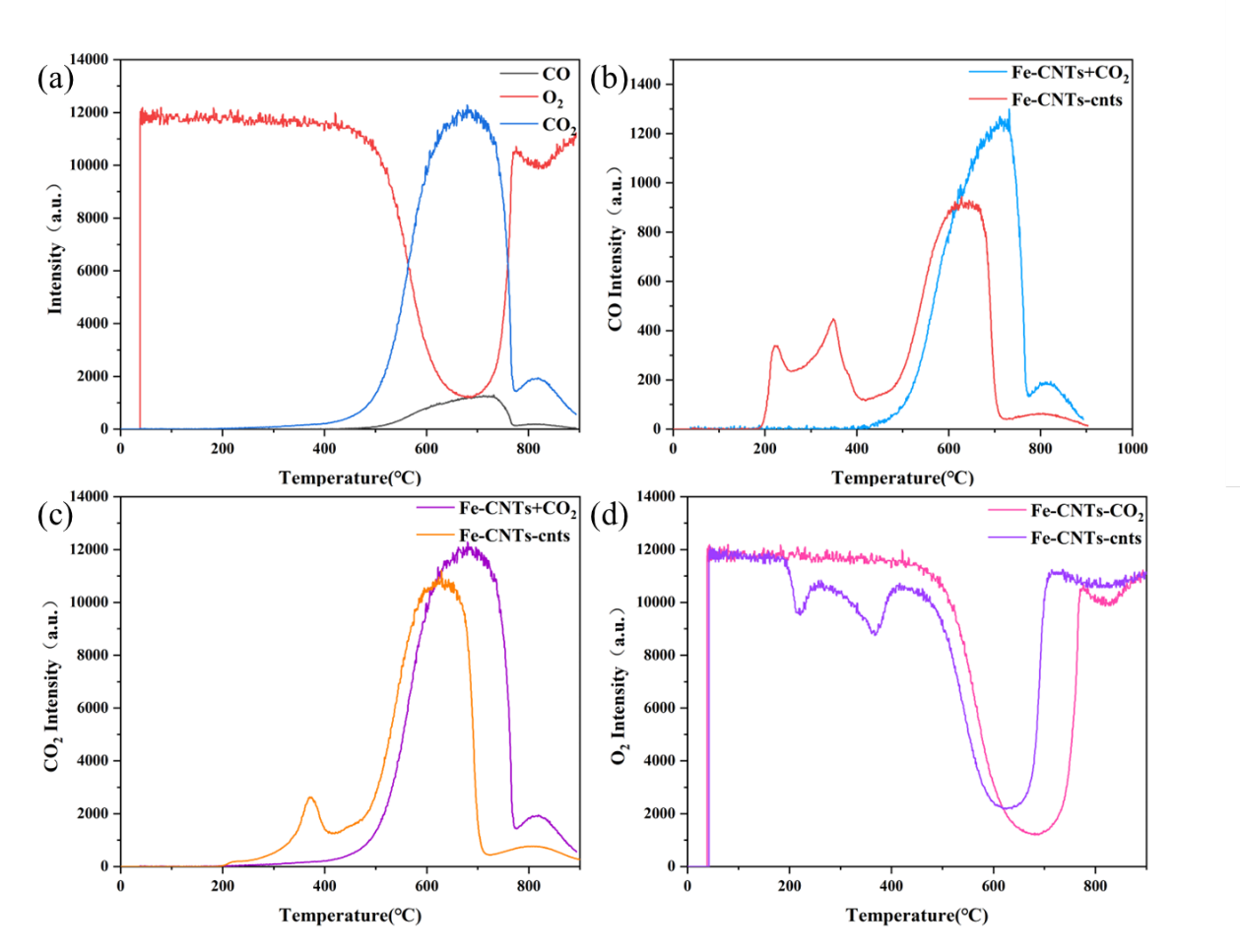


**Fig. S30** TPO results of Fe anchored commercial CNTs (Fe-CNTs-cnts) and CNTs obtained from PP pyrolysis and CO_2_ reforming (Fe-CNTs-CO_2_). (a) A distinct CO_2_ desorption peak is observed at 679.7 °C in the temperature-programmed O₂ reaction (TPR-O₂) profile. (b) O_2_ temperature-programmed oxidation (O₂-TPO) of iron-doped and CO₂-treated carbon nanotubes (CNTs) shows a rightward shift of (b) the CO generation peak, (c) CO_2_ generation peak and (d) O_2_ consume peak, reflecting an elevated reaction temperature relative to pristine Fe-CNTs.

The ST2742C four-probe system was employed to measure the electrical conductivity of CNTs obtained after microwave pyrolysis and CO_2_ reforming under an applied pressure range of 2–30 MPa, as shown in Figure S30. At 30 MPa, commercial carbon nanotubes exhibited the electrical conductivity at 36.406 S/cm, while the CNTs after PP pyrolysis CO_2_ reforming reached 44.114 S/cm.


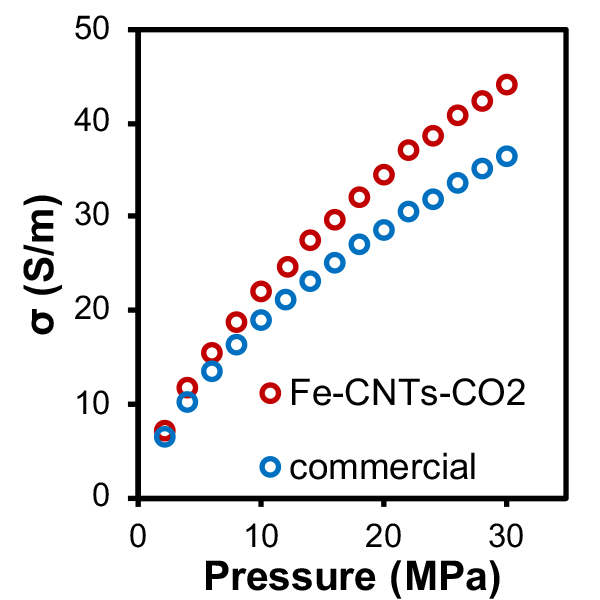


**Fig. S31** Electrical conductivity test results of Fe anchored commercial CNTs and CNTs obtained from PP pyrolysis and CO_2_ (Fe-CNTs-CO2).

6.5 Performance of Fe-gap@CNTs in microwave assisted pyrolysis


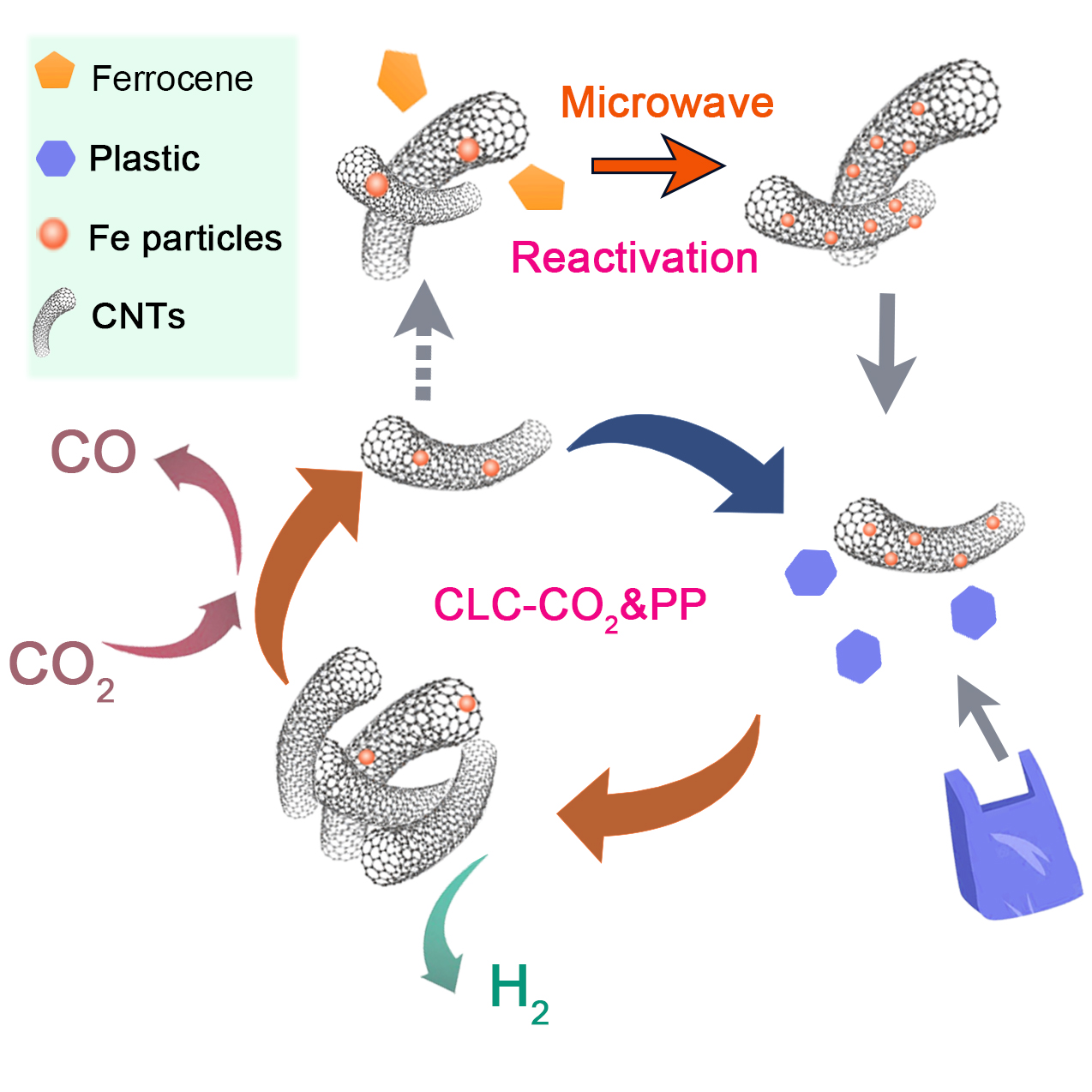


**Fig. S32** Concept design of close-loop conversion of greenhouse gas and waste plastic to syngas assisted with dual-function catalyst reactivation


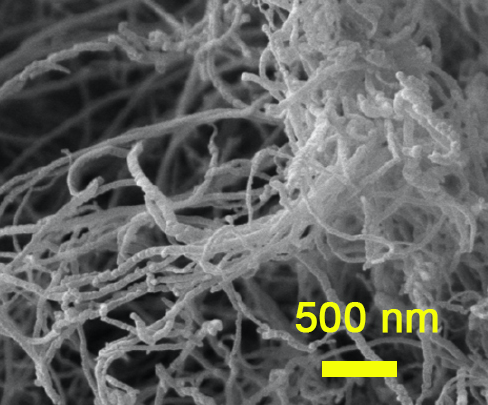


**Fig. S33** SEM images of CNTs obtained from PP pyrolysis catalyzed by Fe-gap@CNTs.


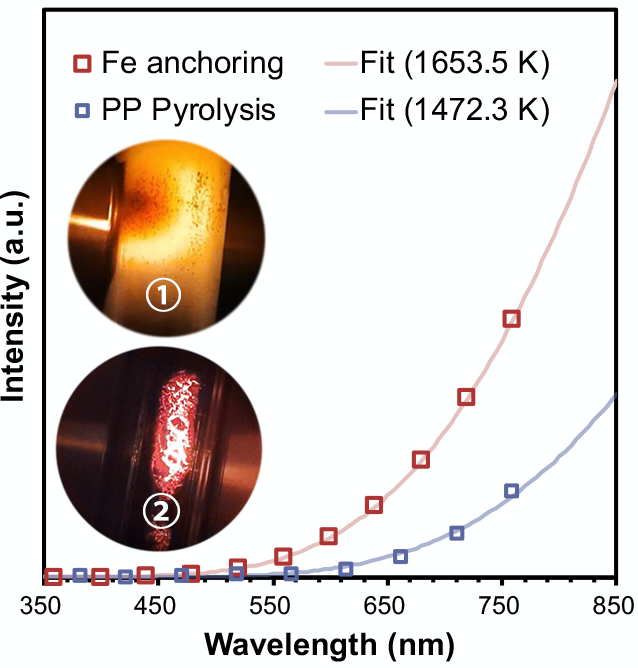


**Fig. S34** Fitted results of emitted luminescence from PP-catalyst mixture irradiated by microwave.

**Fig. S35** Reactor wall’s maximal temperature measured by IR camera. (Red domain represents the sample is irradiated by microwave).

**Fig. S36** Comparison of heating rate and oil yield.

6.6 Applicability of microwave induced carbon removal

To validate the applicability of microwave induced carbon removal, we repeated the microwave induced ultrafast heating process by using petroleum coke produced by a refinery factory in Shijiazhuang (Hebei province, China). We mix these industrial carbon wastes with the pretreated iron anchored CNTs to initiate ultrafast heating under microwave irradiation. Upon switching on microwave generator, the mixed sample emit strong yellow light within seconds, indicating the achievement of high temperatures. Subsequently, the generation of CO was monitored by the gas analyzer. After 13 minutes, the microwave generator was turned off. The total mass reduction of petroleum coke reached above 58%.


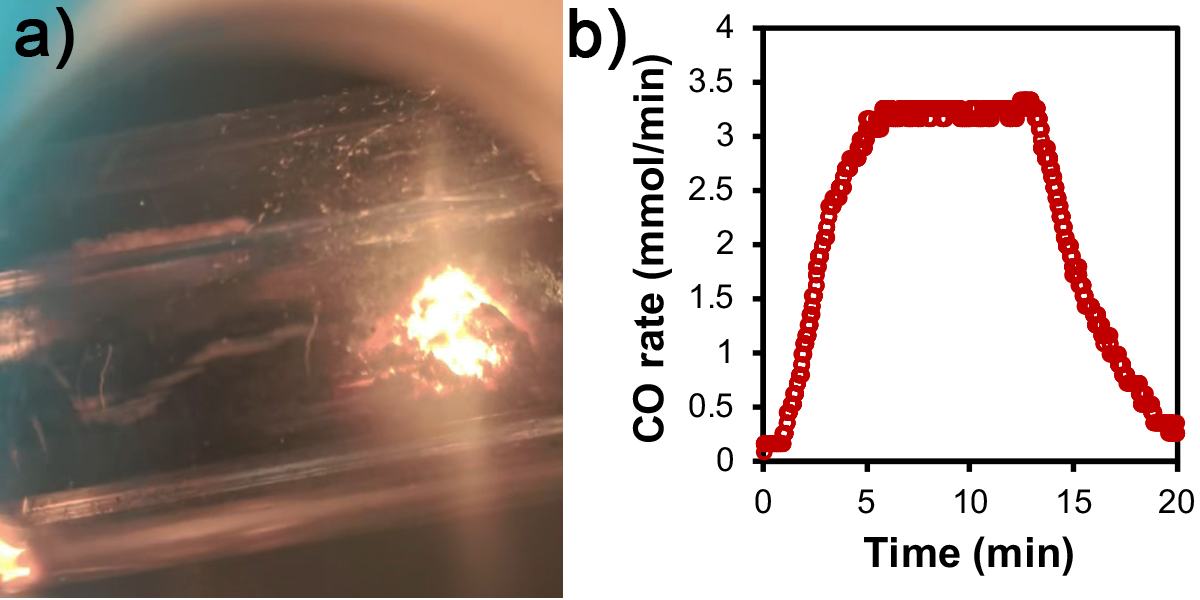


**Fig. S37** Carbon removal from petroleum coke via Boudouard reaction induced by microwave. (a) Experimental phenomenon during microwave heating. (b) Generation rate of CO during microwave irradiated coke removal.

**7. Technical and economic analysis (TEA)**

7.1 Description of unit operations

Carbon Capture

The CO₂ absorption capture process mainly includes absorption and regeneration, supported by pretreatment, heat recovery, and product treatment. In pretreatment, flue gas is cooled, purified, and pressurized to meet absorption requirements. During absorption, cleaned flue gas countercurrently contacts lean solvent in the absorber, forming CO₂-rich solvent. After CO₂ removal, the purified flue gas is vented. In regeneration, rich solvent is heated or purged to release CO₂ and regenerate lean solvent. Key steps like solvent circulation and heat recovery reduce energy consumption and enable solvent reuse. CO₂ product is purified and compressed for storage or utilization.

The core equipment of the absorption-based CO₂ capture system includes absorbers, regenerators, heat exchangers, pumps, and compressors. Absorbers and regenerators, often packed or tray towers, facilitate gas-liquid contact for CO₂ absorption and desorption. Heat exchangers recover heat to preheat rich solvent, reducing energy use. Reboilers or purge devices provide heat for CO₂ desorption. Coolers and condensers remove heat and purify CO₂. Pumps and fans ensure solvent circulation and flue gas flow. Auxiliary equipment like water wash towers and separators enhance system efficiency. Taking MEA-based chemical absorption as an example, the process involves CO₂ absorption by MEA in the absorber and desorption in the regenerator. The required equipment is listed in Table S5.

**Table S5** Equipment for carbon capture units

|  | Specifications | Number | Capital cost/10^4^$ |
| --- | --- | --- | --- |
| Absorption column, vessel | φ6 × 15，0.2 MPa | 2 | 90.34 |
| Desorption column, vessel | φ4 × 10，0.2 MPa | 2 | 42.36 |
| Absorption column, vessel | φ6 × 15 | 2 | 32.37 |
| Desorption column, vessel | φ6 × 15 | 2 | 11.51 |
| Absorption column, reboiler | 1500 m^2^ | 2 | 131.57 |
| Absorption column, condenser | 1500 m^2^ | 1 | 101.09 |

Pressure Swing Adsorption (PSA)

The purification of carbon monoxide via pressure swing adsorption (PSA) relies on cyclical operation of multiple adsorption beds to achieve continuous separation. The process comprises the following 7 stages: (a) Adsorption, during which feed gas is introduced into the adsorption bed (0.1–2.5 MPa). The selective adsorption of CO (or impurity components such as CO₂/H₂S) occurs, while weakly adsorbed species (e.g., H₂, N₂) are eluted from the bed outlet. (b) Pressure equalization, during which post-adsorption beds are hydraulically connected to regeneration beds to establish pressure equilibrium. This step facilitates gas recovery (e.g., CO) and enhances the gas recovery efficiency. The number of pressure equalization stages can be optimized (typically 6–23 cycles) depending on specific process requirements. (c) Cocurrent depressurization after pressure equalization to release partial adsorbed gas, which serves as purging medium for adjacent beds. (d) Countercurrent depressurization to atmospheric/negative pressure against the adsorption direction enables desorption of strongly adsorbed components (CO₂/H₂S), thereby achieving preliminary adsorbent regeneration. (e) Purge/Displacement with weakly adsorbed gas (e.g., H₂) or pure CO further eliminates residual impurities to improve adsorbent regeneration efficacy. (f) Optional vacuum pumping reduces bed pressure to negative values (e.g., 0.099 MPa) to intensify desorption and minimize impurity content (e.g., CO₂) in the desorbed gas stream. (g) Final Pressurization, during which the bed is repressurized to adsorption conditions using product/feed gas to prepare for the subsequent adsorption cycle.

To achieve the above steps, the required equipment mainly includes: (1) Adsorption column filled with adsorbents, with the quantity determined by process design (e.g., 5~32 towers), enabling alternating adsorption and regeneration. (2) Buffer Tank, including feed gas buffer tank, product gas buffer tank, cocurrent depressurization/countercurrent depressurization gas buffer tank, etc., which stabilize gas pressure and flow rate (e.g., 50~200 m³). (3) Compressor / Vacuum pump. (4) Programmable Valves for controlling the step switching of each bed. (5) Control System for monitoring parameters such as pressure, flow rate, and temperature in real time, and automatically adjusting timing and valve actions. In the adsorption column, 5A molecular sieve is used for separating CO from N₂ and CH₄, while Cu⁺-loaded molecular sieve is used to selectively adsorbs CO through π-complexation. The required equipment is listed as Table S6.

**Table S6** Equipment for PSA units

|  | Specifications | Number | Capital cost/10^4^$ |
| --- | --- | --- | --- |
| Adsorption, vessel | φ1000 × 12050，4.4 MPa | 8 | 91.19 |
| Mixed gas Buffer tank for mixed gas | φ1000 ×5650 | 18 | 111.77 |
| Roots blower | Exhaust rate 20m^3^/min | 6 | 4.28 |
| Other compressors | Exhaust pressure 4.0 MPa | 3 | 12.84 |
| Vacuum pump | Extraction rate 1200 L/S | 15 | 10.65 |

Hydrogen purification

The technology for purifying hydrogen involves a combination of membrane separation and cryogenic distillation processes. The feed gas, which is the tail gas from PSA with most CO removed and contains 1.7 t/h of hydrogen along with small amounts of CO and N₂, is first processed at 35℃ and 5 bar. It is compressed to 10 bar by compressor K-1 and then cooled to 25℃ through cooler E-1. The treated gas then undergoes a three-stage membrane separation process. Membranes Mem-1 and Mem-2 work to enrich hydrogen, resulting in a hydrogen purity of 99.96% and a recovery rate of 95.87% at the outlet of Mem-2. Following membrane separation, the hydrogen enters cold box CB-1. Here, through sublimation at -61℃ and 4 bar, the residual CO₂ solidifies, which purifies the hydrogen to 99.999% while also pre-cooling it for subsequent liquefaction. The pre-cooled hydrogen at -61℃ and 4 bar is further cooled to the liquefaction temperature of -252℃ via two cryogenic heat exchangers (CHX-2 and CHX-3). After undergoing ortho-para conversion in balance reactors (ER-1 and ER-2) and pressure reduction to 1.3 bar by expander T-5, saturated liquid hydrogen (LH₂) is finally obtained. According to literature estimates, the cost of the membrane separation unit is 0.203 $/kg H₂, and the cost of the liquefaction unit is 0.92 $/kg H₂.

7.2 Calculation results of TEA

**Table S7** Summary of product sales revenue (100 kton)

| Product | Output (t) | Market price ($/t) | Revenue (million $) |
| --- | --- | --- | --- |
| H_2_ | 12960 | 1507 | 19.53 |
| CO | 270320 | 536 | 144.89 |
| Low defect CNTs | 22032 | 50000 | 881.28 |
| **Total** |  |  | **1100.80** |

* All TEA were calculated in RMB and converted to US dollars at the exchange rate of August 5, 2025.

* Considering the realities of industrial production, tenaciously extending the reaction time to achieve maximum aromatic yield is not sensible. Therefore, we have set the retention time of PP to 0.5 hour under microwave irradiation while this value should be extended to 4 hours in the traditional routes.

**Table S8** Summary of product costs (100 kton)

|  | **Item** | **Quantity per year (10^4^ t)** | **Cost per unit (USD)** | **Calculated value (10^4^ USD)** |
| --- | --- | --- | --- | --- |
| Raw materials | | | | |
| 1 | Waste PP | 10 | 278.4/t | 2784.00 |
| 2 | CO_2_ | 26.93 | 48.59/t | 1308.53 |
| 3 | ferrocene | 0.24 | 983/t | 235.92 |
| 4 | adsorbents | 0.0375 | 9400/t | 352.5 |
| **Utilities** | | | | |
| **1** | Refrigerant | 1260.6 kWh | 11.84/kWh | 14926 |
| **2** | Electricity | 18216 kWh | 0.1/kWh | 1821.6 |
| **3** | Hot oil | 1583.2 kWh | 1.11/kWh | 1757 |
| **4** | Water | 2155040 GJ | 0.354/GJ | 76 |
| **5** | Low-pressure steam | 2155040 GJ | 7.72 $/GJ | 1663 |
| **Total** |  |  |  | **24924.6** |

* Low-pressure steam 1: 125 °C; Low-pressure steam 2: 175 °C; Medium-pressure steam: 250 °C

* The cost of the catalyst is primarily composed of niobium oxide, ZSM-5, and Ru. Each ton of catalyst contains 0.66 tons of niobium oxide, 0.33 tons of ZSM-5, and 6.6 kg of Cu, resulting in an estimated unit price of $9400 per ton. Given the adsorbent's stability, the annual usage is projected to be 375 tons.

**Table S9** Key assumptions about capital investment (200 kton)

|  | Cost(10^4^ $) | Note |
| --- | --- | --- |
| **Fixed capital investments** | **38773.0** |  |
| *(1) Direct cost* | *26671.0* |  |
| Costs of major equipment | 10459.2 |  |
| Microwave equipment | 5633.4 |  |
| pyclone separator | 4.2 |  |
| pump | 1.9 |  |
| reactor | 4172.2 |  |
| tank | 118.97 |  |
| heat exchanger | 232.6 |  |
| column and plate | 267.77 |  |
| blower and compressor | 27.77 |  |
| Other direct costs | (specified as % of equipment cost) | |
| Installation | 2091.8 | 20% |
| Piping | 2091.8 | 20% |
| Instrumentation and control | 2091.8 | 20% |
| Building & structure | 3137.8 | 30% |
| Yard improvement | 1045.9 | 10% |
| Service facilities | 5229.6 | 50% |
| Land | 523.0 | 5% |
| *(2) Indirect cost* | *12002.0* |  |
| Engineering & supervision | 4000.7 | 15% |
| Legal expenses | 533.4 | 2% |
| Construction expenses | 4000.7 | 15% |
| Contractor's fee | 1600.3 | 3% |
| Contingency | 2667.1 | 10% |
| **Working capital** | **5816.0** | 15% of fixed capital investment |
| **Others** | **15529.7** |  |
| Depreciation period | 10 yr |  |
| Annual depreciation rate | 0.1 |  |
| Depreciation charge | 1045.9 |  |
| Annual interest rate | 3% |  |
| Operating labor | 2492.5 | 10% of total product cost |
| Operating supervision | 124.6 | 5% of operating labor |
| Utilities | 3738.8 | 15% of total product cost |
| Maintenance and repairs | 2667.1 | 10% of fixed capital investment |
| Operating supplies | 800.1 | 30% of maintenance and repairs |
| Laboratory charges | 24.9 | 20% of operating labor |
| Royalties | 997.0 | 4% of TPC without depreciation |
| Plant overhead costs | 1146.3 | 5% of total product cost |
| General expenses | 2492.5 | 10% of total product cost |

**Table S10** Key assumptions about profits (100 kton)

|  | Cost(10^4^ $) | Note |
| --- | --- | --- |
| Variable cost | 60118.7 | see table S8 |
| Salaries | 6011.9 | 10% of variable cost |
| Depreciation | 1045.9 | See table S9 |
| Maintenance and repairs | 1163.2 | 3% of fixed capital investments |
| Insurance | 271.4 | 0.7% of fixed capital investments |
| Total cost | 68611.1 |  |
| Annual profit | 80719.7 | sales revenue in table S7 |
| Business income tax | 24215.9 | 30% of annual profit |
| Net profit | 56503.8 |  |

References

[1] D. X. Luong, K. V. Bets, W. A. Algozeeb, M. G. Stanford, C. Kittrell, W. Chen, R. V. Salvatierra, M. Ren, E. A. McHugh, P. A. Advincula, Z. Wang, M. Bhatt, H. Guo, V. Mancevski, R. Shahsavari, B. I. Yakobson, J. M. Tour, “Gram-scale bottom-up flash graphene synthesis”, *Nature* **2020**, *577*, 647-651.

[2] X. Chen, R. Lu, C. Li, W. Luo, R. Yu, J. Zhu, L. Lv, Y. Dai, S. Gong, Y. Zhou, W. Xiong, J. Wu, H. Cai, X. Wu, Z. Deng, B. Xing, L. Su, F. Wang, F. Chao, W. Chen, C. Xia, Z. Wang, L. Mai, “Activating inert non-defect sites in Bi catalysts using tensile strain engineering for highly active CO<sub>2</sub> electroreduction”, *Nature Communications* **2025**, *16*.

[3] X. Shen, Z. Zhao, H. Li, X. Gao, X. Fan, “Microwave-assisted pyrolysis of plastics with iron-based catalysts for hydrogen and carbon nanotubes production”, *Materials Today Chemistry* **2022**, *26*.

[4] A. I. Persson, M. W. Larsson, S. Stenström, B. J. Ohlsson, L. Samuelson, L. R. Wallenberg, “Solid-phase diffusion mechanism for GaAs nanowire growth”, *Nature Materials* **2004**, *3*, 677-681.

[5] D. V. Suriapparao, R. Vinu, “Resource recovery from synthetic polymers via microwave pyrolysis using different susceptors”, *Journal of Analytical and Applied Pyrolysis* **2015**, *113*, 701-712.

[6] D. V. Suriapparao, A. Yerrayya, G. Nagababu, R. K. Guduru, T. H. Kumar, “Recovery of renewable aromatic and aliphatic hydrocarbon resources from microwave pyrolysis/co-pyrolysis of agro-residues and plastics wastes”, *Bioresource Technology* **2020**, *318*, 124277.

[7] D. V. Suriapparao, G. Nagababu, A. Yerrayya, V. Sridevi, “Optimization of microwave power and graphite susceptor quantity for waste polypropylene microwave pyrolysis”, *Process Safety and Environmental Protection* **2021**, *149*, 234-243.

[8] S. Fan, Y. Zhang, L. Cui, Q. Xiong, T. Maqsood, “Conversion of Polystyrene Plastic into Aviation Fuel through Microwave-Assisted Pyrolysis as Affected by Iron-Based Microwave Absorbents”, *ACS Sustainable Chemistry & Engineering* **2023**, *11*, 1054-1066.

[9] V. Mortezaeikia, O. Tavakoli, “Understanding the kinetics of waste plastic catalytic pyrolysis under microwave irradiation for enhanced resource valorization”, *Chemical Engineering Journal* **2024**, *500*, 157228.

[10] P. H. M. Putra, S. Rozali, M. F. A. Patah, N. N. N. Ghazali, R. Ahmad, A. Idris, “Effect of metal powder and coil on microwave pyrolysis of mixed plastic”, *Chemical Engineering Journal* **2024**, *487*.

[11] Y. Zhang, C. Yu, X. Song, X. Tan, W. Li, S. Liu, X. Zhu, S. Cui, Y. Xie, J. Qiu, “Defect-enabled local high-temperature field within carbon to promote in-plane integration of an electrocatalyst for CO<sub>2</sub>-to-CO conversion”, *Energy & Environmental Science* **2025**, *18*, 1331-1342.
